# Somatic sex-specific transcriptome differences in Drosophila revealed by whole transcriptome sequencing

**DOI:** 10.1186/1471-2164-12-364

**Published:** 2011-07-14

**Authors:** Peter L Chang, Joseph P Dunham, Sergey V Nuzhdin, Michelle N Arbeitman

**Affiliations:** 1 Section of Molecular and Computational Biology, Department of Biological Sciences, University of Southern California, Los Angeles, California 90089, USA; 2 Department of Biomedical Sciences, College of Medicine, Florida State University, Tallahassee, Florida 32306, USA

**Keywords:** Drosophila, sex hierarchy, genomics, transcriptome, expression

## Abstract

**Background:**

Understanding animal development and physiology at a molecular-biological level has been advanced by the ability to determine at high resolution the repertoire of mRNA molecules by whole transcriptome resequencing. This includes the ability to detect and quantify rare abundance transcripts and isoform-specific mRNA variants produced from a gene.

The sex hierarchy consists of a pre-mRNA splicing cascade that directs the production of sex-specific transcription factors that specify nearly all sexual dimorphism. We have used deep RNA sequencing to gain insight into how the Drosophila sex hierarchy generates somatic sex differences, by examining gene and transcript isoform expression differences between the sexes in adult head tissues.

**Results:**

Here we find 1,381 genes that differ in overall expression levels and 1,370 isoform-specific transcripts that differ between males and females. Additionally, we find 512 genes not regulated downstream of *transformer *that are significantly more highly expressed in males than females. These 512 genes are enriched on the × chromosome and reside adjacent to dosage compensation complex entry sites, which taken together suggests that their residence on the × chromosome might be sufficient to confer male-biased expression. There are no transcription unit structural features, from a set of features, that are robustly significantly different in the genes with significant sex differences in the ratio of isoform-specific transcripts, as compared to random isoform-specific transcripts, suggesting that there is no single molecular mechanism that generates isoform-specific transcript differences between the sexes, even though the sex hierarchy is known to include three pre-mRNA splicing factors.

**Conclusions:**

We identify thousands of genes that show sex-specific differences in overall gene expression levels, and identify hundreds of additional genes that have differences in the abundance of isoform-specific transcripts. No transcription unit structural feature was robustly enriched in the sex-differentially expressed transcript isoforms. Additionally, we found that many genes with male-biased expression were enriched on the × chromosome and reside adjacent to dosage compensation entry sites, suggesting that differences in sex chromosome composition contributes to dimorphism in gene expression. Taken together, this study provides new insight into the molecular underpinnings of sexual differentiation.

## Background

Based on several whole genome sequencing projects it is evident that many genomes contain far fewer genes than were originally predicted based on the apparent complexity of the animals under study. For example, the human genome is estimated to have some 25,000 genes [1,2], which is not that many more than the ~20,000 and ~14,000 genes predicted to be in the genome of the roundworm *Caenorhabditis elegans *or the fruit fly *Drosophila melanogaster*, respectively [3,4]. Additional complexity in form and function is specified by the generation of multiple protein isoforms from individual genes through the production of specific transcript isoforms. Differences in the abundance, and the temporal and spatial expression patterns of transcript isoforms contribute to animal diversity and complexity. Isoform-specific transcripts can arise from different molecular mechanisms, including alternative promoter usage, alternative pre-mRNA splicing, alternative polyadenylation or alternative mRNA degradation [reviewed in [5]]. Determining the repertoire and abundance of isoform-specific transcripts in a temporal- and tissue-specific manner is an important next step towards understanding how each protein isoform contributes to development, physiology and disease. This is greatly facilitated by using new deep sequencing technologies that provide information about the repertoire of isoform-specific transcripts present in biological samples. Here, we sequenced the Drosophila transcriptome from adult head tissues to understand how somatic sex differences are established.

**Figure 1:**
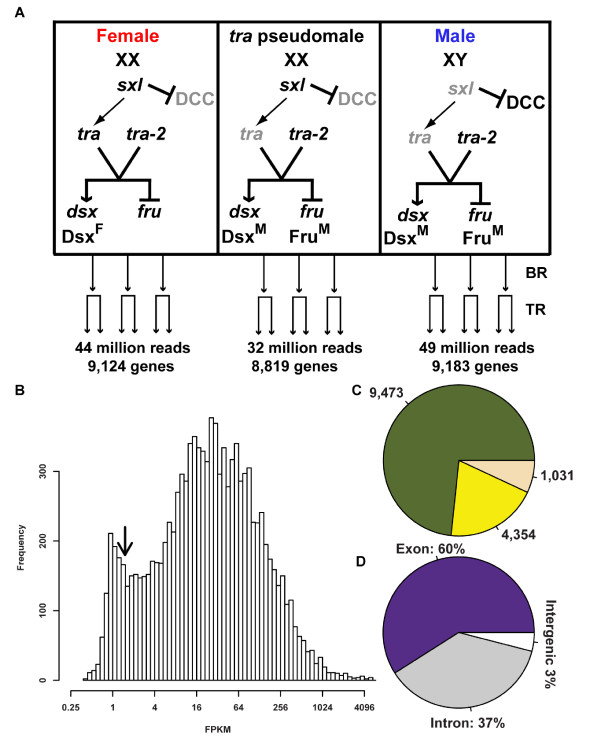
**Experimental Design and Sequence Read Mapping (A) Illumina reads were sequenced and mapped from libraries generated from Drosophila female, male, and *tra *pseudomale head tissues**. For each genotype, there are three independent biological samples, which were sequenced with two technical replicates. The sex hierarchy gene activity and sex chromosome composition for each genotype is shown. Grey indicates that no functional protein is made. The dosage compensation complex (DCC) is not active in females and *tra *pseudomales that have two × chromosomes. The number of sequence reads and genes that the reads map to are shown. The number of biological (BR) and technical replicates (TR) are indicated. (B) FPKM distribution of all genes covered by sequence reads (green in C). Arrow at lower tail of distribution indicates approximately where FPKM values are for *dsx *and *fru*. (C) Illumina reads mapped to 9,473 genes (green) with FPKM values of at least 1in all six replicates within at least one genotype. There were 4,354 genes (yellow) that had reads mapped to the gene, but not with FPKM greater than 1 in all six replicates within at least one genotype. There were 1,031 genes (beige) that had no reads mapped to the gene. (D) Illumina reads were mapped to exons (60%, purple), introns (37%, grey), and intergenic regions (3%; white).

One of the best-studied genetic regulatory hierarchies is the *Drosophila *sex hierarchy, which specifies all aspects of somatic sexual differentiation (Figure 1) [reviewed in [6]]. Females have two × chromosomes, whereas males have an × and a Y chromosome; this difference is the primary signal that establishes differences between the two sexes. In females, the presence of two × chromosomes leads to the production of *sex lethal *(*sxl*) protein from transcripts produced from an early promoter [reviewed in [7,8]]. Later in development, SXL regulates the splicing of *sxl *and *transformer *(*tra*) pre-mRNAs that are expressed in both males and females, resulting in the production of functional SXL and TRA in females and not in males. *tra *encodes a pre-mRNA splicing factor that [9,10], together with the product of *transformer-2*, regulates the splicing of *fruitless *(*fru*) and *doublesex *(*dsx*) pre-mRNAs [reviewed in [6]].

Alternative splicing of *fru *and *dsx *pre-mRNAs results in the production of male-specific FRU isoforms and male- and female-specific DSX isoforms [11-13]. *fru *and *dsx*, at the bottom of the hierarchy, both encode transcription factors that regulate all major aspects of somatic sexual differentiation, with the exception of dimorphism in body size, which is regulated downstream of *sxl*, but not *tra, dsx and fru*. *dsx *specifies nearly all aspects of sexual dimorphism outside the nervous system and also specifies dimorphism within the nervous system [reviewed in [6,14]] [15-18]. *fru *is necessary and sufficient for specifying the potential for nearly all aspects of male courtship behaviors [reviewed in [19]].

SXL also regulates the process of dosage compensation, which is the mechanism that yields roughly the same amount of transcript from the single × chromosome in males to that of the two × chromosomes in females [reviewed in [20]]. Dosage compensation is not active in females because SXL binds to the 3' untranslated region of the *male-specific lethal 2 *(*msl-2*) mRNA to prevent translation [21,22]. MSL-2 is a component of the dosage compensation complex (DCC) that is required for binding to the × chromosome and thus, male-specific MSL-2 production leads to DCC binding to the × chromosome in a male-specific manner. Binding of DCC to the × chromosome in males leads to a less compact × chromosome structure that facilitates increased transcription [reviewed in [20]].

There have been several efforts to identify somatic gene expression differences between males and females that are regulated by the sex determination hierarchy using genomic approaches, including by early subtractive cDNA hybridization approaches [23,24], and later serial analysis of gene expression and microarray approaches [25-29]. While new insights have been gained based on these studies, differences in the transcriptome between males and females could be regulated at several different levels and these previous techniques did not have the resolution to robustly detect many types of differences on a genome-wide scale.

For example, while some genes have differences in overall expression levels regulated downstream of *dsx *and *fru *sex-specific transcription factors, as we have previously shown [27-29], sex-specific differences in expression of low-abundance transcripts would not have been readily identifiable. Additionally, for genes that have multiple promoters, *dsx *and *fru *might regulate one or a subset of a gene's promoters, resulting in dimorphism in the abundance of isoform-specific transcript classes (transcript isoforms), which has been shown for a subset of genes that are sex-differentially expressed [30,31], but has not been determined on a genome-wide level. Given that the top of the sex hierarchy includes genes that function in splicing, it is also possible that there are additional pre-mRNA transcripts that are alternatively spliced in a sex-differential manner by *sxl *and/or *tra *products, or by other sex-differentially expressed splicing factors, such as *CG3056 *(*sxl *paralog) [29], though sex-differences in mRNA splicing have not been determined on a genome-wide scale.

Here we compare gene expression and isoform transcript differences between males, females and *tra *mutant animals in adult head tissues to gain insight into how the sex hierarchy establishes sex-specific differences. *tra *regulates the splicing of *dsx *and *fru*, which are both expressed in adult head tissues and specify sexual dimorphism in the nervous system and adult head fat body, the two major tissues of the adult head. Using the Illumina GAI sequencing platform, we identify 1,381 genes and 1,370 transcript isoforms with sex-differential abundances, and a set of 362 genes that have dimorphism in the ratio of isoform-specific transcripts. A large set of genes with male-biased expression that are not regulated downstream of *tra *are located on the × chromosome, with many adjacent to entry sites for the DCC, suggesting that the dosage compensation process also contributes to dimorphism in transcript abundance. Nearly as many genes have transcript isoform dimorphism as those that have overall abundance dimorphism, suggesting that differences in isoform-specific transcript expression is an important way to generate sexual dimorphism, using a limited repertoire of genes. For genes with significant differences in the ratio of transcript isoforms between the sexes, we could not detect robust significant differences in a set of transcription unit features, relative to all transcript isoforms expressed in head tissues, demonstrating that there is likely not a single molecular mechanism that generates dimorphism. Additional genome-wide computational searches using the TRA and SXL binding motifs revealed additional potential targets of these sex hierarchy pre-mRNA splicing factors.

## Results

The complexity, identity and abundance of the mRNA population in Drosophila head tissues was determined using the Illumina GAI sequencing platform to comprehensively determine sex-specific differences in mRNA abundance. RNA was derived from 0-24 hour adult male and female heads of the Berlin wild type strain and from chromosomally XX *tra *pseudomale heads. *tra *encodes a pre-mRNA splicing factor in the Drosophila somatic sex determination hierarchy that is required in females for sexual development (Figure 1). *tra *pseudomales look and behave almost identically to wild type males, though they are larger in body size and are sterile because they lack germline tissues. Illumina sequencing libraries were generated from purified polyA mRNA. For each genotype, three independent biological samples were assayed, each with two Illumina sequencing performance replicates, such that six replicates per genotype were sequenced. The data was mapped onto the complete Drosophila genome (Release 5.29; 14,858 predicted genes), using the Tophat software program and computationally analyzed together to provide the greatest depth of coverage [32]. Only reads that mapped to one unique region between the 5' and 3' limits of the annotated transcripts for a gene, including the potential splice junctions and intron sequences, were utilized for further gene coverage analyses.

### Mapping Illumina RNA-sequence reads to Drosophila genomic sequence

The pooled data from all three genotypes had a total of 125 million (M) mapped reads (44 M from female, 49 M from male, and 32 M from *tra *pseudomales) that were 36 bases long, resulting in ~128 fold average coverage of annotated genes (Additional file 1). Genes that were confidently covered by sequence analyses here are those for which all six replicates within at least one genotype have FPKM (fragments per kilobase per million sequenced reads) values of at least 1, including sequence reads that map to untranslated regions (UTR) and intron sequences, which totaled 9,473 genes (64% of all annotated genes) (Figure 1 and Additional file 2). Overall, the number of genes that were covered by sequence reads in female (9,124), male (9,183) and *tra *mutant (8,819) head tissues were similar. Using this criterion, we find that 61%, 62%, and 59% of annotated genes had transcripts present in adult male, female and *tra *mutant head tissues, respectively (Additional file 2). To assess the quality of the sequencing data, the reproducibility of the results was assessed for each genotype and shows a high degree of reproducibility among replicates (R^2 ^ranges from 0.93 to 0.96; Additional files 3, 4 and 5).

There were 5,385 genes that were not expressed in any genotype, and 1,031 genes among these 5,385 genes for which there were no reads that mapped to the gene (no reads in all 18 libraries; see Additional file 6). Given the high sensitivity afforded by deep-sequencing techniques, the absence of detecting reads for many of these genes likely reflects either a true absence of expression or that transcripts were present at very low amounts. This list of genes contain 1,664 genes with known significant high expression in the testis, as determined by Flyatlas [33] through the Flymine portal [34], which is consistent with these genes not having high expression in head and/or somatic tissues. This set of 5,385 genes was also enriched [33,34] with genes that encode products with functions in DNA repair, including the pathways *ATM mediated phosphorylation of repair proteins *(*P *< 1.2e-18; 17 genes), and *Homologous Recombination Repair *(*P *< 9.9e-3; 35 genes) [33], suggesting that these DNA repair pathways are not highly active in young adult head tissues (Additional file 7). The set of 5,385 genes was enriched on chromosome arm 2L (Hypergeometric Test for all chromosome bias analyses; *P *< 3.5e-5) and depleted from the × chromosome (*P *< 6.0e-7) and chromosome arm 3R (*P *< 0.0025) (Additional file 8 for chromosome bias analyses).

Overall, there is variability in the number of reads that map across the transcription units of many genes [35,36]. It has been shown that the use of random hexamer priming for library preparation results in biases that influence the uniformity of the coverage along transcription units [35]. We compared the coverage along the coding regions of genes, normalized based on the coverage of the entire gene, and found higher variability at the 5' and 3' ends of the transcription unit, though this coverage at the ends was not significantly higher or lower than the mean coverage along the length of transcript (Additional file 9). It is not unexpected to have more variability in the number of sequence reads from the ends of a transcription unit [reviewed in [37]]. Coverage of sequence from introns is increasingly higher on average as the reads are approaching the 3' end of the transcription unit, which is expected if splicing occurs in a 5' to 3' direction along the transcription unit, as the transcript is being produced.

Approximately 60% of the total mapped reads were from annotated exon sequences, whereas the remaining mapped reads were from annotated intron (37%) and unannotated intergenic sequences (3%) (Figure 1). The observation that such a large number of reads map to intron sequences suggests that the polyA mRNA fraction analyzed contained some immature transcripts. Nevertheless, because mapping was done onto the complete genome using uniquely mapped reads, the data provides a snapshot of an enriched polyadenylated mRNA pool within head tissues, captured at several processing stages. In addition, several sequenced reads mapped to ribosomal genes (16%), genes that encode tRNAs (2%), as well as small RNA genes.

Genes that are known to produce rare transcripts in adult head tissues, based on RNA blot analyses, have detectable sequenced reads here. For example, *fru *and *dsx*, two sex hierarchy genes that are thought to produce rare transcripts that are detectable by RNA blot [11,12], have FPKM values of 1.67 and 1.43, respectively, which in the distribution of FPKM values for genes in this study is towards the low end (Figure 1). This demonstrates that deep-sequencing is a sensitive approach, even for detecting low-abundance transcripts. Data for all genes is provided in the Additional files.

### Gene transcript abundances differences between females and males

To identify gene expression differences between males and females we limit the analyses to reads that map to exon sequences, as opposed to the full gene sequence that contains introns. We use the term gene expression differences here to describe differences in gene transcript abundance for simplicity, but we note that differences in transcript abundance may be due to other processes, such as differential mRNA stability and degradation, in addition to expression level differences. A gene is considered expressed if all six replicates have FPKM values of at least 1, resulting in 8,561 (58%), 8,594 (58%) and 8,397 (57%) genes that were expressed in female, male, and *tra *mutant head tissues, respectively (Table 1). This is consistent with the ~60% estimated to be expressed in adult heads using microarray techniques [34]. We found that 8,128 genes were expressed in all three genotypes. All subsequent analyses on differential gene expression were limited to the 8,896 genes that were expressed in at least one genotype (Additional file 10).

**Table 1:** Genes that are expressed

Genotype	Genes Expressed
Female	8,561

Male	8,594

*tra *pseudomales	8,397

**Figure 2:**
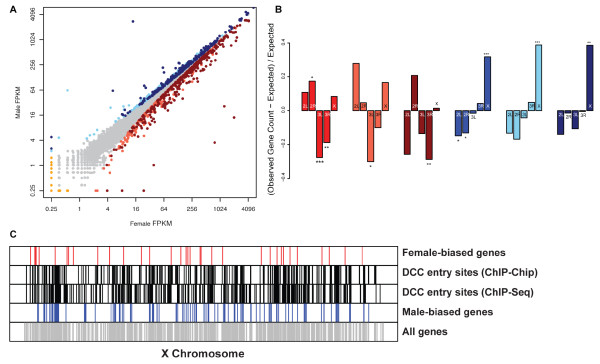
**Genes with overall sex differential expression and their chromosome distribution**. (A) Dot plot showing comparison of gene expression in female and male. Genes with significantly higher expression in male (blue), upstream of *tra *(light blue) or downstream of *tra *(dark blue) are indicated. Genes with significantly higher expression in female (red), upstream of *tra *(light red) or downstream of *tra *(dark red). Yellow dots indicate genes with expression in only female or male genotype. (B) Bar plot showing chromosomal enrichment of genes with female-biased (red), female-biased expression upstream of *tra *(light red), female-biased expression downstream of *tra *(dark red), male-biased expression (blue), male-biased expression upstream of *tra *(light blue), and male-biased expression downstream of *tra *(dark blue). Asterisks indicate significant over- or under-enrichment at three different significance levels (P < 0.05, 0.01 and 0.001 are indicated by *, **, and ***, respectively). (C) Distribution of genes with female-biased (red), or male-biased (blue) expression, within DCC-bound regions (black), or expressed genes among any genotype (gray) along the × chromosome are shown. The DCC-bound regions include those found by ChIP-Chip and ChIP-seq studies [40,41].

To determine if there are genes with differences in expression between the genotypes, an exact test analogous to the Fisher's exact test was implemented on read counts through edgeR in the Bioconductor statistical package [38]. We identified 1,381 genes with differential expression between males and females (*q *< 0.05). Of these, 566 and 815 genes were found to have higher expression in female and male head tissues, respectively (Figure 2, Table 2 and Additional file 10). The genes identified here with sex-differential expression show concordance with our previous microarray study examining sex-specific differences in transcript abundance in head tissues from Berlin animals [28]. For the 566 genes identified with female-biased expression here, 510 had data in our microarray experiments. Of the 510 genes, 219 had significant female-biased expression (*q *< 0.5; a reduced stringency cut-off was used given microarray data tend to have higher variance). Of the 815 genes with male-biased expression here, 684 had data in our microarray experiments. Of the 684 genes, 194 genes had significant male-biased expression (*q *< 0.5). As expected with the increased sensitivity and lower variance in the data using the Illumina platform [39], here we are able to detect more genes with significant differences.

**Table 2:** Genes sex differentially expressed

	Female-biased genes	Male-biased genes
Sex-differentially expressed*	566	815

Upstream of *tra*^#^	203	512

Downstream of *tra^^^*	199	206

* *q *< 0.05 in female-male comparison

# *q *< 0.05 in female-male comparison and *q *> 0.2 in female-*tra *comparison

^ *q *< 0.05 in female-male comparison and *q *< 0.2 in female-*tra *comparison, with the expression bias in the same direction in both comparisons.

The data was examined to determine how many genes have sex-differential transcript abundance regulated downstream of *tra*. Of the 566 and 815 genes with significant female- and male-biased expression, respectively, 199 and 206 genes were regulated downstream of *tra*, having female- or male-biased expression in the female-male comparison as well as having female- or *tra*-biased expression in the female-*tra *comparison (*q *< 0.2). *tra *mutants are pseudomales, and so genes with male-biased expression that are regulated downstream of *tra *should have *tra*-biased expression (Figure 2 and Additional file 10). Even if we use a more stringent statistical criterion to identify genes regulated downstream of *tra *(*q *< 0.05), there are similar numbers of genes identified, with 198 and 195 genes having female- and *tra*-biased expression, respectively. In our previous microarray study, a large fraction of genes found to have sex-differential expression (754 genes) were also not regulated downstream of *tra *(117 genes; 15% of genes with sex-differential abundance) [28].

As expected, the set of genes with sex-differential expression not regulated downstream of *tra *includes *male-specific lethal 2 *(*q *< 8.3e-6 in male-female comparison and *q *= 1 in female-*tra *comparison; male-biased gene) and *RNA on the × 2 *(*q *< 9.8e-97 in male-female comparison and *q *= 1 in female-*tra *comparison; male-biased gene), which are genes in the sex hierarchy regulated upstream of *tra *and involved in dosage compensation (Figure 1). *RNA on the × 1 *(*roX1*) is not included in this set, even though it is known not to be regulated downstream of *tra*, due to mapping of a small number of reads from both female and *tra *head tissues, relative to male tissues. *roX1 *shows the expected large and significant fold-difference between males and females (*q *= 0; average FPKM is 2,786 in males and 13.5 in females), with females and *tra *pseudomales showing a significant difference in reads, but with very few sequence reads detected from *tra *and female animals, as expected (*q *< 1.6e-5; FPKM is 3.7 in *tra *mutants). No sequenced reads mapped to *tra *from RNA derived from *tra *animals. Additionally, genes that are expected to be regulated downstream of *tra *were identified, including *Yolk protein 1, Yolk Protein 2*, and *Yolk protein 3 *(for all three *Yolk protein *genes *q *= 0 in both female-male and female-*tra *comparisons; female-biased gene), and *Cyp4D21 *(*q *< 0.05 in both female-male and female-*tra *comparison; male-biased gene).

### Analysis of genes with sex differential expression and their chromosomal position

Genes with sex-differential mRNA expression might differ in expression due to sex-specific chromosome composition, which would include genes that have differences in expression in the male-female comparison, but not in the female-*tra *comparison. This is because females are chromosomally XX, while males are chromosomally XY; in the female-*tra *pseudomale comparison, all animals are chromosomally XX (Figure 1). In Drosophila, the amount of transcripts produced from the × chromosome is equalized between males and females by up-regulating expression of genes on the single × chromosome in males to roughly equal the amount produced from the two × chromosomes in females [reviewed in [20]].

We identified 203 and 512 female- and male-biased genes, respectively, that are not regulated downstream of *tra *(Additional file 10). These 512 male-biased genes are significantly overrepresented on the × chromosome (116 genes; *P *< 1.7e-4; Additional file 8), which is expected if the differential expression is due to the dosage compensation process up-regulating expression of genes on the single male × chromosome more than two-fold, which may in turn alter expression on other chromosomes and could account for the additional 396 genes that are male-biased and not regulated downstream of *tra*. This bias is similar to our observation using microarray approaches [28]. Male-biased genes that were found downstream of *tra *showed a more moderate overrepresentation on the × chromosome (48 genes; *P *< 7.6e-3), compared to male-biased genes regulated upstream of *tra*. Genes with female-biased expression not regulated downstream of *tra *were underrepresented on chromosome arm 3L, though they were not under or overrepresented on the × chromosome (Figure 2).

Next we determined if genes with sex-biased expression were enriched or depleted from chromosomal regions known to be bound by the dosage compensation complex (DCC) along the × chromosome. Given that the DCC complex modifies chromatin structure in males so that the male × chromosome is less tightly packed and more accessible for transcription, it would be predicted that genes with male-biased expression might reside adjacent to DCC-bound regions and genes with female-biased expression would not. Previous studies have identified entry sites for DCC binding to the × chromosome (hereafter called DCC-bound regions) and their associated genes using chromatin immunoprecipitation approaches, where the DCC-complex is immunoprecipitated in different mutant backgrounds that facilitate the identification of entry sites and then the associated DNA is detected by DNA microarray (CHiP-Chip) or sequence analyses (CHiP-seq) [40,41].

Sex-differentially expressed genes that reside adjacent to DCC-bound regions were ~4 × (82 and 19 genes with male-and female-biased expression, respectively; CHiP-Chip) and ~2 × (52 and 31 genes with male-and female-biased expression, respectively; CHiP-seq) more likely to have male-biased than female-biased expression. When considering only genes that are sex-differentially expressed, not regulated downstream of *tra *and adjacent to DCC-bound regions, genes were ~20 × (56 and 3 genes with male-and female-biased expression, respectively; CHiP-Chip) and ~4 × (34 and 8 genes with male-and female-biased expression, respectively; CHiP-seq) more likely to have male-biased than female-biased expression. Furthermore, when considering genes regulated downstream of *tra *and those not downstream of *tra*, the majority of genes with male-biased expression that reside adjacent to DCC-bound regions were regulated upstream of *tra *(56 vs. 23 genes, CHiP-Chip, and 34 vs. 14 genes, CHiP-seq), whereas this trend was reversed for genes with female-biased expression (3 vs. 6 genes, CHiP-Chip, and 8 vs. 13 genes, CHiP-seq), which is expected if sex-specific expression differences upstream of *tra *are due to dosage compensation.

We next compared the ratio of male- to female-biased genes within DCC-entry site bound regions (ChIP-chip) to the ratio of male- to female-biased genes across the entire × chromosome. We found 82 and 19 genes with male- and female-significantly-biased expression, respectively, within the DCC-bound regions (ratio of 4:1), which is significantly more male-biased than the ratio of male- to female-biased expression throughout the entire × chromosome (ratio of 7:4) (*P *< 2e-6). If we only consider the genes significantly male-biased and regulated upstream of *tra*, we see a similar result, with more male-biased genes residing near the DCC-bound regions (19:1; *P *< 3e-6). When we do a similar analysis with the ChIP-seq data we do not find a significant difference in the ratios, as the ratio of male- to female- significantly-biased genes within DCC-bound regions is similar to the × chromosome-wide ratio (*P *< 0.64). The ChIP-chip and ChIP-seq studies were performed using different tissue sources and so the difference in our comparisons to the two studies could be due to tissue-specific DCC-binding differences [40,41]. Taken together, there is a clear relationship between male-biased genes that are regulated upstream of *tra *and their residence on the × chromosome, with additional evidence suggesting that these genes reside adjacent to DCC-entry sites.

Interestingly, genes with female- and male-biased expression that were not regulated downstream of *tra *do not appear to be from a random set with respect to GO functional enrichments. Genes with female-biased expression not regulated downstream of *tra *were enriched with genes known to be expressed in the adult fat body, larval fat body, and mated and virgin spermatheca tissues, whereas genes with male-biased expression not regulated downstream of *tra *were enriched with genes with known expression in the nervous system, including adult eye, brain, larval CNS, and ventral nerve cord tissues, as assessed using the Flymine web portal analyses of Flyatlas data [33,34]. It is important to note that *tra *pseudomales do not make germline tissues and therefore expression differences observed between wild type males and females that are due to gene expression changes in male head tissues that are male-germline-dependent would not be observed in *tra *pseudomales-female comparisons. These genes would also appear to have sex differential expression upstream of *tra*.

### Gene transcript isoform abundance differences between females, males and *tra *pseudomales

Next we identified genes with sex-differences in transcript isoform abundance ratios, by using Cufflinks to assemble the mapped reads and to estimate the expression of individual transcript isoforms [32,42]. While this program does not report the assigning of reads to a transcript isoform, it does estimate the amount of transcript isoform expression based on these reads, using the gene models from Flybase. Given that Flybase annotation has some inherent biases, we also perform *de novo *annotation of genes (see below). From this analysis using known gene models, we found that 1,711 genes among the 8,896 expressed genes produce more than one transcript isoform, among the genotypes examined here (Additional file 11). To validate the measures of transcript isoform expression predictions by Cufflinks, we determined our expected number of junction reads based on the transcript isoform abundance models of Cufflinks, and compared that number to the number of reads that directly map to annotated junction sequence, as detected by Tophat. Using the FPKM number calculated by Cufflinks, we calculated the expected number of reads that should span each junction by summing the FPKM's that are predicted for each junction. We obtained linear relationships between expected and counted reads in log scale with R^2 ^values between 0.70 and 0.74, confirming that the transcript isoform FPKM values found using Cufflinks correlate well with the number of junctions mapped by Tophat (Additional file 12). These R^2 ^values are consistent with expectation, considering that not all sheared cDNA fragments that span a junction contain sequence data for the junction (i.e. in cases where a fragment spans a junction, but both paired end sequence reads only map to exon sequences that do not contain the junction); these reads would be assigned to a junction by Cufflinks but would not count as reads that map to a junction by Tophat.

A transcript isoform was considered expressed if all six replicates had FPKM values of at least 0.5. We next identified genes with sex-differential transcript isoform ratios as genes for which the ratio of isoform expression in one sex is different from the ratio in the other sex (Table 3 and Additional file 11). We identified 362 genes (*q *< 0.05) with sex-differences in the ratio of transcript isoform expression. There were 263 among these genes that were regulated downstream of *tra *(*q *< 0.2). We find that similar to gene expression, there was an overrepresentation of genes from the × chromosome that have sex-differential isoform expression ratios (81 genes; *P *< 0.007), though this was not true when only considering genes regulated downstream of *tra *(55 genes; *P *< 0.11) (Additional file 8).

**Table 3:** Genes with sex biased ratios of transcript isoforms

	Genes
Sex-differential ratios	362

Downstream of *tra*	263

Next, we examined the number of transcript isoforms with sex-differences in overall abundance among the 4,368 transcript isoforms produced from the 1,711 genes with at least two isoforms. We found that 486 and 884 transcript isoform have female- or male-biased expression differences (*q *< 0.05), with 279 and 328 having female- and male-biased expression regulated downstream of *tra *(*q *< 0.2), in the same direction as expected from the female-male comparison (Table 4). From the 1,370 transcript isoforms with sex-differential expression, 708 transcript isoforms are from genes that showed sex-differential expression at the gene level (see above). Here, we identify 662 transcript isoforms from 474 genes that did not show sex-differential expression at the gene level. This demonstrates the increased power to identify genes that underlie sexual dimorphism, by having the ability to detect transcript isoform differences.

**Table 4:** Isoform transcrips differentially expressed

	Female-biased	Male-biased
Sex-differentially expressed*	486	884

Upstream of *tra*^#^	153	439

Downstream of *tra^^^*	279	328

* *q *< 0.05 in female-male comparison

# *q *< 0.05 in female-male comparison and *q *> 0.2 in female-*tra *comparison

^ *q *< 0.05 in female-male comparison and *q *< 0.2 in female-*tra *comparison, with the expression bias in the same direction in both comparisons.

**Figure 3:**
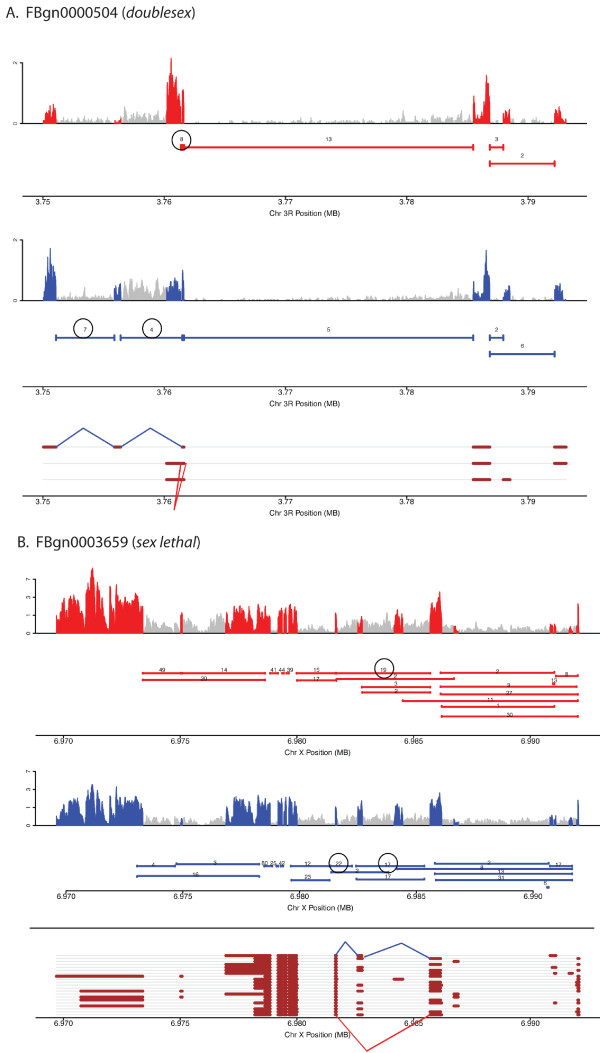
**Coverage plots, junction plots and gene models for genes with sex-differential transcript isoforms, data for (A) *doublesex *and (B) *sex lethal *is shown**. Throughout red and blue indicate data from female and male, respectively. Coverage plots for exon sequences are shown with peaks in red and blue indicating coverage from RNA from females and males, respectively; grey indicates non-exonic gene regions as annotated by Flybase. Junction plots are shown as solid horizontal lines beneath the coverage plots. The number above each line indicates the number of sequence reads that span a junction. All numbers are based on 1 million mapped reads. Flybase gene models are shown at the bottom of each panel with exon regions shown in brown. Female- and male-preferred junctions are indicated by red and blue lines between donor and acceptor sites on the gene models. The circled numbers in the junction plots correspond to the female and male preferred junctions.

**Figure 4:**
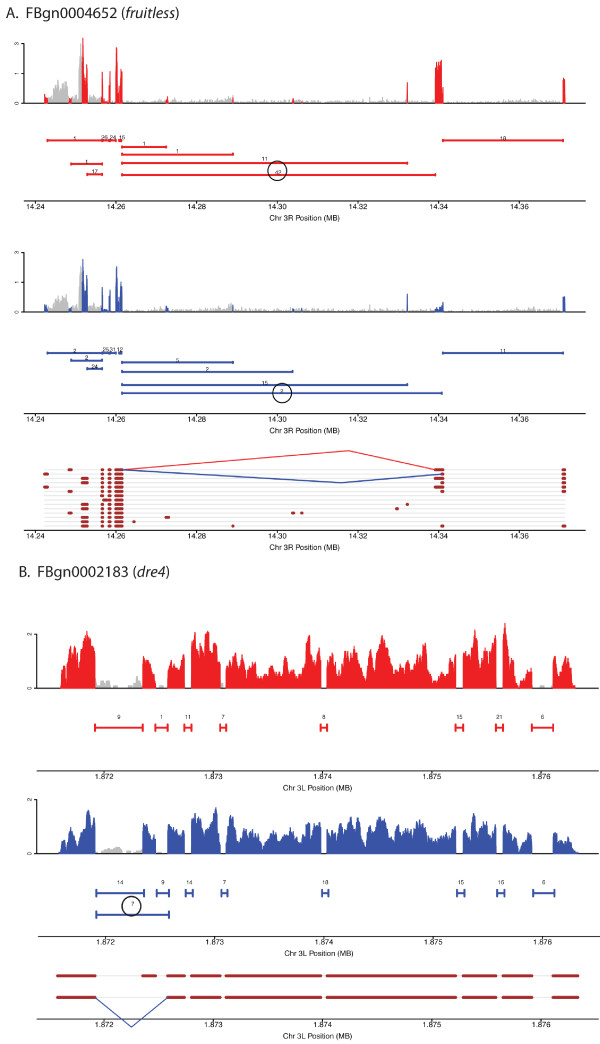
**Coverage plots, junction plots and gene models for genes with sex-differential transcript isoforms, data for (A) *fruitless *and (B) *dre4 *is shown**. Throughout red and blue indicate data from female and male, respectively. Coverage plots for exon sequences are shown with peaks in red and blue indicating coverage from RNA from females and males, respectively; grey indicates non-exonic gene regions as annotated by Flybase. Junction plots are shown as solid horizontal lines beneath the coverage plots. The number above each line indicates the number of sequence reads that span a junction. All numbers are based on 1 million mapped reads. Flybase gene models are shown at the bottom of each panel with exon regions shown in brown. Female- and male-preferred junctions are indicated by red and blue lines between donor and acceptor sites on the gene models. The circled numbers in the junction plots correspond to the female and male preferred junctions.

**Figure 5:**
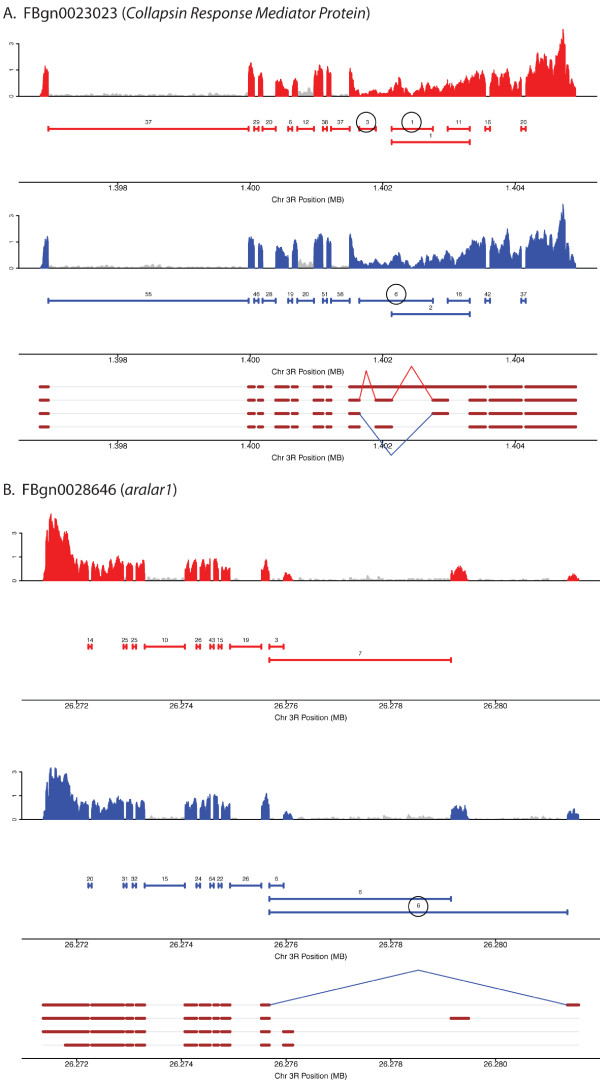
**Coverage plots, junction plots and gene models for genes with sex-differential transcript isoforms, data for (A) *Collapsin Response Mediator Protein *and (B) *aralar1 *is shown**. Throughout red and blue indicate data from female and male, respectively. Coverage plots for exon sequences are shown with peaks in red and blue indicating coverage from RNA from females and males, respectively; grey indicates non-exonic gene regions as annotated by Flybase. Junction plots are shown as solid horizontal lines beneath the coverage plots. The number above each line indicates the number of sequence reads that span a junction. All numbers are based on 1 million mapped reads. Flybase gene models are shown at the bottom of each panel with exon regions shown in brown. Female- and male-preferred junctions are indicated by red and blue lines between donor and acceptor sites on the gene models. The circled numbers in the junction plots correspond to the female and male preferred junctions.

The fact that we observe a similar number of transcript isoform with sex-differences, compared to the number of genes with overall gene expression abundance differences, suggests that a primary mechanism to generate sex-differences in head tissues is through the use of alternative gene isoforms. This is particularly noteworthy considering that these differences were found in 1,711 genes that expressed more than one isoform in this study, whereas 8,896 genes were analyzed for overall abundance differences. Genes with sex-biased transcript isoform abundance include *sex lethal *(FBtr0100206, FBtr0100208, FBtr0300840), *doublesex *(FBtr0081760, FBtr0081761, FBtr0081759), and *fruitless *(FBtr0083642, FBtr0083644, FBtr0083648, FBtr0083641), as expected (see Figure 3, 4 and 5).

We also wanted to determine if there are additional genes that show robust sex-differences in transcript isoform abundances, regulated downstream of the sex hierarchy. For this analysis, we required that a gene have more than one transcript isoform, that the transcript isoform have significant (*q *< 0.05) and substantial differences (Fold difference > 2) in both the female-male and the female-*tra *pseudomale comparison, in the same direction. We find 44 and 93 transcript isoforms with robust female- and male-biased expression differences downstream of *tra*, respectively (Additional file 13). For many of the genes that encode these transcript isoform, the ratio of transcript isoform are also significantly different between the sexes, with 40 and 77 genes that have female- and male-biased transcript isoform abundances also showing a significant difference in the ratio of transcript isoform abundance between the sexes (*q *< 0.05). Though we do identify transcript isoforms that show as substantial a difference in levels between males and females and females and *tra *pseudomales, as we see for sex hierarchy genes *dsx*, *fru*, *sxl *and *tra*, we do not see an enrichment of TRA or SXL binding sites in most of these transcript isoforms (see below, *Psa *is one exception), suggesting that the robust difference is not due to alternative splicing by these sex hierarchy splicing factors.

The 44 transcript isoforms with robust female-biased expression include those encoded by genes in the sex hierarchy (*fru*, *sxl *and *dsx*). Transcript isoforms that show female-specific expression include: *sluggish A *(*slgA*; FBtr0077210), which is involved in locomotor behavior and phototaxis and encodes a product with proline dehydrogenase activity; *dsx *(FBtr0081760); and *TER94 *(FBtr0088392), which encodes a protein involved in protein localization. The 44 transcript isoforms are encoded by genes that have an enrichment of the following GO terms: cell development (13 genes, *P *< 2.0e-7), nervous system development (10 genes, *P *< 3.2e-5), and sex differentiation (4 genes, *P *< 3.9e-5), as assessed using Flymine [34].

The 93 transcript isoforms with robust male-biased expression include those encoded by genes in the sex hierarchy (*fru *and *dsx*). Transcript isoforms that show male-specific expression include: *trehalase *(FBtr0071537), which encodes an enzyme for trehalose metabolism; *another B-box affiliate *(FBtr0086572), which encodes a product with protein binding functions; and *Vha55*, which encodes a vacuolar H^+ ^ATPase (FBtr0301661) [43]. The 93 transcript isoforms are encoded by genes that have enrichment of the following GO terms: behavior (13 genes; *P *< 1.7e-5), signaling (24 genes, *P *< 1.9e-5), and central nervous system development (15 genes, *P *< 6.6e-4), as assessed using Flymine [34].

### Analysis of mechanisms that generate transcript isoform differences

To gain insight into the mechanisms that generate differences in transcript isoforms abundances between sexes, we analyzed how the isoforms differ. If sex-differential isoform abundance is due to alternative splicing, we expect to find sex-differences in internal cassette and 3' exons, whereas if the difference is due to alternative promoter usage, we would expect to find differences in 5' exons. There are multiple mechanisms to generate transcript isoforms, which includes five major categories [44]. The first mechanism is the inclusion or exclusion of cassette exons, as observed for the sex-specific splicing of *dsx*. *dsx *contains 6 exons; female- and male-enriched mature *dsx *transcripts include exons 1-4 or exons 1-3, 5, and 6, respectively. The second mechanism is through the use of either an alternative 5' donor site, or 3' acceptor site, as observed for sex-specific splicing of *tra*. Here, two alternative acceptor sites exist and determine the male or female gene products. In males, the upstream acceptor site is chosen and results in an elongated exon 2 and premature stop site usage, which produces nonfunctional TRA. In females the downstream acceptor site is used, preventing the production of an elongated exon 2 and results in functional TRA. A third mechanism is the use of alternative promoters and poly-adenylation sites, which switches the most 5' and 3' exons, respectively. The fourth and fifth mechanisms of generating isoform diversity are seen less frequently through the use of retained introns and trans-splicing of two independent mRNA molecules.

We determined if the transcript isoforms from 362 genes with significant sex-differential expression ratios are enriched with any transcription unit structural features. For this analysis we determined if the pair of male- and female-biased transcripts from a gene differ significantly in their 5' or 3' exons, their 5' or 3' exon length, or with the inclusion/exclusion of cassette exons, as compared to a random set of pairs of male- and female-biased transcripts; these latter random pairs were not required to show a significant difference in transcript ratios between the sexes. The random set of pairs is from the 1,711 genes with at least two expressed transcript isoforms. For this analysis we compared the Flybase annotated exon structure to determine how the transcripts in a pair differ, with respect to several features (Table 5).

**Table 5:** Exon structure differences between female- and male- preferred transcript isoforms

1711 Genes with Multiple Isoforms	5prime Exon	5prime Extension	Internal Exon	3prime Extension	3prime Exon
Number of Genes	786	226	562	275	230

Median Length (bases)	240	21.5	196.5	99	544.5

					

**362 Genes with Significant Ratios**	**5prime Exon**	**5prime Extension**	**Internal Exon**	**3prime Extension**	**3prime Exon**

Number of Genes	157	49	120	64	36

*P *value*	0.18	0.43	0.43	0.18	0.018†

Median Length (bases)	194	18	157.5	155.5	725.5

*P *value^+^	0.012†	0.16	0.022†	0.17	0.043††

					

**263 Genes with Significant Ratios downstream*****tra***	**5prime Exon**	**5prime Extension**	**Internal Exon**	**3prime Extension**	**3prime Exon**

Number of Genes	118	36	92	50	26

*P *value*	0.43	0.48	0.21	0.088	0.03†

Median Length (bases)	206	17.5	153.5	467.5	725.5

*P *value^+^	0.094	0.35	0.026†	0.0029††	0.14

					

**90 Genes with Robust Male Isoform Expression**	**5prime Exon**	**5prime Extension**	**Internal Exon**	**3prime Extension**	**3prime Exon**

Number of Genes	35	14	25	24	8

*P *value*	0.11	0.29	0.19	0.0053††	0.13

Median Length (bases)	194	15.5	136	623.5	494.5

*P *value^+^	0.015†	0.45	0.17	0.00067††	0.46

					

**42 Genes with Robust Female Isoform Expression**	**5prime Exon**	**5prime Extension**	**Internal Exon**	**3prime Extension**	**3prime Exon**

Number of Genes	18	4	16	7	3

*P *value*	0.42	0.34	0.27	0.52	0.17

Median Length (bases)	224.5	45	180.5	9	3703

*P *value^+^	0.28	0.13	0.33	0.087	0.0056††

*Wilcoxon Rank-Sum Test

+One-sided Hypergeometric test

**† **indicates underrepresented and **†† **indicates overrepresented.

We find that the most common structural differences among the random pairs of female- and male biased transcript isoforms are those at the 5' end of a gene. We observed that 1,012 isoform pairs differed in their 5' start positions, including 786 pairs that differed by an additional 5' exon (median length: 240 bases); 226 pairs shared a portion of their 5'-most exon, with one transcript isoform having additional bases upstream (median additional bases: 21.5). For 505 pairs that differed in their 3' end positions, 230 pairs differed by an additional 3' exon (median length: 544.5 bases); 275 pairs shared a portion of their 3'-most exon, with one transcript isoform having additional bases downstream (median additional bases: 99). There were 562 pairs that differed with the presence of an internal cassette exon in one of the isoforms (median length: 196.5), with 297 exons having lengths in multiple of 3's.

We next determined if there are any biases with respect to these transcription unit features among the sex-biased transcript isoforms produced from 362 genes with significant sex-differences in isoform ratios and the 263 among these 362 genes that were regulated downstream of *tra*. We only detect a significant decrease in the number of additional 3' exons from these two sets, respectively (*P *< 0.018 and *P *< 0.03; Table 5). Interestingly, we find that the median 5' base differences tend to be shorter than the median 3' base differences for all gene sets, and this is especially pronounced for genes with sex-biased ratios of transcript isoforms (Table 5), though this difference may be due to differences in Flybase annotations of genes, which is richer in the annotation of the 5' end of genes [45]. It should be noted that isoform pairs may differ by more than one annotation feature described above. In addition, isoform pairs that differ at the 3' end may also have differences upstream that may change the coding frame.

We were also not able to detect any significant relationship between sex-differential transcript isoforms abundance with respect to other various gene features. For example, the relative position of an exon or intron within the gene annotation unit and the length of an exon or intron were not correlated with female- or male-biased abundance. In addition, when considering the length of an intron, we were not able to find sex-preferential abundance for a given reading frame (data not shown). These results demonstrate that for sex-differential transcript isoforms there is not a significant difference in constitutive exons, cassette exons, or introns coverage with respect to their location within the annotation unit, length, or reading frame (Additional files 14, 15, 16, 17, 18 and 19).

### Sex differential transcript isoform abundance and the sex hierarchy

Molecular-genetic analyses have demonstrated that nearly all aspects of somatic sexual differentiation are regulated downstream of *tra *in the sex determination hierarchy by directing the alternative splicing of *dsx *and *fru*. As validation of our approach, *dsx *(*q *< 3.4e-24), *fru *(*q *< 4.7e-94), and *sxl *(*q *< 1.4e-127) all had significant sex-differences in the ratio of their isoforms between female and male, which is expected since these genes produce sex-specific transcripts due to alternative splicing. The genes *dsx *(female-*tra *comparison: *q *< 2.3e-24) and *fru *(female-*tra *comparison: *q *< 9.7e-177) also had significant differences in isoform ratios regulated downstream of *tra*, while *sxl *did not (*q *= 1), as expected given the known sex hierarchy regulatory relationships (Figure 1).

To determine if there are additional sex-specific phenotypes that are regulated downstream of *tra *via the RNA splicing function of *tra*, we determined if there are additional genes likely to be regulated directly by TRA. We found 10 genes, among the 1,711 genes that expressed at least two isoforms, that contained the TRA binding site [46] (Additional files 20, 21). Six of these genes were found among the 362 genes with sex-specific differences in transcript isoforms expression ratios (Hypergeometric Test; *P *< 0.0081). As expected, among the six genes were *dsx*, *fru*, and *sxl*; also included in this list were *alan shepard (shep)*, *CG12484*, and *Puromycin sensitive aminopeptidase (Psa)*. *Psa *was among the genes that have robust differences in transcript isoforms abundance, whereas *shep *and *CG12484 *were not. From the computational analyses, it is not clear if TRA has a role in regulating the splicing of these three genes. Future molecular-genetic studies on these genes will be important in determining if they are true targets of TRA.

We also determined if sex-differences in transcript isoform abundance might be downstream of *sxl*. Among the 1,711 genes that expressed more than one isoform, 107 genes contained the SXL binding site [47]. Only 22 of these genes were found among the 362 genes with sex-specific differences in transcript isoform expression ratios between female and male (Hypergeometric Test; *P *< 0.59), suggesting that the SXL binding site was not a significant factor in splicing within this data set. It should be noted that the SXL binding site is not as well characterized as the TRA binding site and does not have a definitive consensus.

### Analysis of gene expression through *de novo *identification of annotations

In addition to gene annotations provided by Flybase, Cufflinks has the option of assembling genes without any *a priori *gene structure information. To identify the set of genes expressed in Drosophila head tissues using *de novo *means with only the genome sequence, we pooled all reads from every sample and used Tophat to map to the genome sequence. Cufflinks was used to assemble these genes and their isoforms. Sequenced reads from each sample were then analyzed independently, assigning reads to genes and isoforms that were identified *de novo*. Using the previous definition of expression where we require all replicates in a genotype to have FPKM values of at least 1, we identified 6,324 genes that were expressed in at least one genotype. The number of genes identified here is less than the 8,896 expressed genes identified using Flybase annotation, mainly because the power of Cufflinks is decreased without the gene annotation. Using known donor and acceptor splice sites, we identified 5,504 genes on Flybase among the 6,324 genes that were identified *de novo *using Cufflinks. We also identified 169 multi-exon genes that were not previously annotated on Flybase, which are found between known annotated genes, as well as 651 multi-exon genes that overlap with previously annotated genes, but do not share donor and acceptor sites (Additional file 22).

## Discussion

Sequencing enriched mRNA fractions from adult male, female and *tra *pseudomale Drosophila head tissues has revealed additional insights regarding gene expression dimorphism, transcript isoform expression dimorphism and sex hierarchy gene regulation. The additional sensitivity of this technique allowed for the identification of 1,381 genes (*q *< 0.05) with overall sex-differential expression, many of which were not identified in our previous microarray study [28]. We further distinguished between regulation upstream and downstream of *tra *for genes with sex differential expression, with the idea that genes regulated upstream of *tra *might have sex differential expression due to differences in sex chromosome composition. For genes that are regulated upstream of *tra*, those with male-biased expression showed a significant enrichment on the × chromosome, as we previously have shown [28]. In this study, the larger set of genes with male-biased expression that reside on the × chromosome, together with the recent identification of DCC entry sites on the × chromosome, afforded the opportunity to analyze if genes with male-biased expression that reside on the × are in close proximity to DCC entry sites. We show that the chromosomal positions of these male-biased genes are adjacent to known DCC-entry sites found in one study, but not the second; it should be noted that these studies used different tissues [40,41]. On the other hand, genes with female-biased expression regulated upstream of *tra *did not show a significant enrichment on the × chromosome, and further, those that do reside on the × chromosome are not likely to be adjacent to known DCC entry sites. In contrast to our finding, a previous study found that genes with male-biased expression are not adjacent to DCC-bound regions [48]. In Bachtrog *et al*. the male-biased genes were identified from gonadectomized flies, gonads and whole animals and sex hierarchy regulation was not considered, which may account for the difference in the results we obtained here.

These results are consistent with the idea that dosage compensation in males leads to × chromosome chromatin being less tightly packed in males, resulting in the higher expression of many genes along the entire single × chromosome in males, to that of the two × chromosomes in females. One possibility is that this may be functional and important to male-specific biology in head tissues, rather than simply a byproduct of dosage compensation acting in a non-precise manner. The idea that increased expression might be functional is bolstered by the fact that genes with no expression in the adult head were significantly depleted from the × chromosome (*P *< 2.1e-10; Additional file 8), suggesting that the × chromosome is more permissive for gene expression than other chromosomes and might harbor unique classes of genes where this increased expression level might be functional. Genes that need to be tightly regulated, with no leaky expression, might be selectively removed from the × chromosome. However, one alternative explanation is that DCC complex binds preferentially to active genes, as has been shown [reviewed in [49]], which is consistent with the observation that genes with nervous system function are enriched in the male-biased set considered here, since head tissues are enriched with genes that are expressed in the nervous system. Thus, the increased expression might not be functional, but reflects that some tissues can tolerate gene expression dosage differences among all of the chromosomes. Future studies that examine sex-biased gene expression in other tissues will help distinguish between these possibilities, since if the latter hypothesis is true, it is expected that genes with tissue-specific expression that reside on the × chromosome will have higher expression in males in the tissue that they are expressed within.

It is also important to consider the quantitative genetic and evolutionary implications of the 'overcompensation' of gene expression in males. If it is simply a mechanistic by-product of being too close to DCC entry sites, then the 'overcompensation' might be mal-adaptive in males. If so, these genes could evolve generally weaker expression levels, reducing their expression in males, but potentially resulting in 'under-expression' in females. These patterns appear plausible from our analyses of genetic variation among natural Drosophila genotypes and comparisons between sexes [50]. There, sex determination genes were expressed at higher levels in some genotypes, in both males and females; or were expressed at lower levels in other genotypes, again both in males and females. Furthermore, the impossibility of reaching gene expression levels on the × chromosome that result in the highest fitness levels in both sexes generates trade-offs between the sexes. These trade-offs have been extensively documented in many studies and have shown that when × chromosomes have been maximized for male fitness, this was costly in females, and the other way around [51]. Alternatively, the 'overcompensation' could potentially be adaptive; it is then likely uncoupled to the expression level in females at the level of population variation. Additionally, it has previously been hypothesized that the overall expression variation in males has a simpler genetic basis than in females (Wayne *et al*. 2007). We hypothesize that the hemizygous state of male × chromosome might primarily explain this simpler pattern and might further contribute to the more simple variation of autosomal gene expression level observed in males, which are under *trans *control of the hemizygous male × chromosome genes. The autosomal genes that are expressed more strongly in males than in females thus might be under the positive control of 'overcompensated' × chromosome genes. Testing these hypotheses will be one future direction of our research.

Examination of transcript isoform differences between males and females demonstrated that the production of alternative protein isoforms is likely to be an important mechanism to generate sexual dimorphism. Nearly as many genes show sex differences in overall gene expression differences, as is observed for transcript isoform abundance differences (1,381 genes and 1,370 isoform-specific transcripts). With respect to how differences in transcript isoform abundances are generated, no robust enrichment of a set of transcription unit structural features were observed, relative to the distribution of these features in all known transcript isoforms. For example, transcript isoforms that showed sex-differential abundances did not have a significant enrichment of differences in their internal cassette exons, or 5' exons, relative to all transcript isoforms, though a moderately significant difference was detected for 3' exons. Computational analyses of the genes with sex-differential transcript isoform abundance has led to the identification of three additional genes that might be regulated at the level of alternative pre-mRNA splicing directed by *tra*; however two of these genes only show weak quantitative differences in transcript isoform ratios between the sexes, with *Psa *showing significant sex-specific transcript isoform ratio differences. Future molecular studies will bear on whether the pre-mRNA transcripts from these three genes are targets of TRA.

The observation that there are so many genes with sex differences in transcript isoform abundances may explain our previous difficulty in identifying genes regulated downstream of the sex-specific transcription factors encoded by *dsx *and *fru *in our previous microarray studies, which did not have the resolution to detect isoform-specific transcripts. For example, in our previous studies, we found many genes that showed sex differences in transcript abundance downstream of *tra*, but we could not place them downstream of *dsx *or *fru*. In hindsight, this may be because there are sex-specific differences in the ratio of a gene's transcript isoforms or in transcript isoform abundance that would not have been detected. Perhaps for many genes regulated downstream of *dsx *and *fru*, it is at the level of isoform-specific transcripts and not overall gene expression. This makes sense when considering that the Drosophila genome is compact and that this compact structure might confer some evolutionary advantage [reviewed in [52]]. If maintaining a compact genome is important, it might be better to confer sex-specific regulation on an existing gene, which might also have non-sex-specific functions, by adding a new promoter, exon, or intron structure to the locus, while maintaining the non-sex-specific functions through different isoform-specific transcripts. Further studies examining transcript isoform expression in *fru *and *dsx *mutants will begin to address these outstanding questions. It will also be important to examine gene expression differences in small sets of cells, rather than entire head tissues.

While alternative pre-mRNA splicing is the key mechanism to direct sex-specific development and physiology at the top of the sex hierarchy, it appears not to be a primary mechanism to influence sexual differentiation downstream of *tra *function. In this study, we did not find a significant and robust enrichment of a set of transcription unit features in transcript isoforms with dimorphism in abundance, relative to all transcript isoforms, nor did we find many additional genes with robust dimorphism in transcript isoform abundance downstream of *tra *and that contain *tra *binding sites, as is observed for genes in the sex hierarchy. Based on this observation, it appears that most sexual dimorphism downstream of *tra *is established at the transcriptional level downstream of *dsx *and *fru*, or by undiscovered mechanisms of sex hierarchy gene function. For example, *sxl *was first shown to encode a product with pre-mRNA splicing functions, acting on *sxl *and *tra *pre-mRNAs [reviewed in [7,53]]. Later, it was discovered that SXL also acts to regulate *msl-2*, by functioning as an mRNA binding protein, but not for pre-mRNA splicing, but rather to prevent translation of *msl-2 *in females. After these studies, SXL was then shown to function in the hedgehog signaling pathway to influence the stability of the complex that acts downstream of the receptor and ultimately the size difference observed in males and females [54]. In the coming years, it will be interesting and exciting to uncover the mechanisms that regulate sexual dimorphism downstream of the sex hierarchy in both a functional and evolutionary context.

## Conclusions

In this study we identified 9,473 genes expressed in adult head tissues when we mapped sequence reads to the entire annotated gene, including intergenic regions, and 8,896 genes expressed if we mapped sequence reads to exon sequences. Of these 8,896 genes, 1,381 genes showed sex differential expression, but only a fraction of these genes were regulated downstream of *tra*. Examination of the genes with sex-biased expression regulated upstream of *tra *revealed that male-biased genes are enriched on the × chromosome and reside adjacent to dosage compensation entry sites. Using the algorithm Cufflinks, which assigns sequence reads to isoform transcripts, we show that 362 genes have sex-differences in the ratio of transcript isoform expression and that 1,370 transcript isoforms have overall sex-differential expression levels. Of these 1,370 isoform transcripts, only 708 are from genes that show overall sex-differential expression. No robust enrichment of transcription unit structural features was detected in genes with sex-biased expression, suggesting that no single molecular mechanism accounts for the production of sex differences in isoform transcripts.

## Methods

### Drosophila stocks and head tissue collection

Flies were raised at 25°C under a 12-hour light and 12-hour dark cycle on standard cornmeal food media. The wild type flies were the Berlin strain. Chromosomally XX, *tra *pseudomales are the genotype *y,w*, *P[w^+mC^, ubi-gfp]/w*; *tra^1^/Df(3L)st-j7*. Chromosomal males and females were distinguished as follows: chromosomally XX flies did not have white eyes, as they received an × chromosome that expressed white (*P[w^+cM^, ubi-gfp]) *from their fathers, whereas chromosomally XY flies had white eyes, as they received the Y chromosome from their fathers. Flies were collected 0-24 hours after eclosion as follows. Fly bottles were cleared at 5 pm. At 9 am the following day, adults were anesthetized using CO_2_, separated by sex, and kept in food vials to recover from the stress of the CO_2 _treatment. At 5 pm that same day, flies were transferred by gentle tapping into a cryovial and snap frozen in liquid nitrogen. Flies were stored at -80°C until enough were collected for each biological replicate (~200 flies).

Adult heads were snapped off from the body by shaking the frozen flies in the cryovial. The frozen heads were sorted from the bodies on plastic cooled on dry ice. Total RNA was extracted from ~200 heads per sample, using 1 ml of Trizol (Invitrogen). The head tissue was homogenized in Trizol using a motorized homogenization drill and total RNA was extracted.

### Sample Illumina library preparation

RNA purification, cDNA synthesis and Illumina library construction were performed using the protocols of Mortazavi *et al*. [36], with the following modifications. Total RNA, mRNA, and DNA were quantified using a Qubit fluorometer (Invitrogen). mRNA fragmentation was performed using Fragmentation Reagent (Ambion) for a 3 minute and 50 second incubation at 70°C and subsequently cleaned through an RNA cleanup kit (Zymo Research). Additional DNA and gel purification steps were conducted using Clean & Concentrator kits (Zymo Research).

Three biological head Illumina library replicates were generated for male, female, and *tra*-mutant genetic backgrounds for paired-end 36-base pair reads. Each biological replicate was sequenced twice on separate lanes on an Illumina Genome Analyzer I, producing a total of six replicates per genotype.

### Sequence Alignment and Algorithm

Sequencing reads were aligned and mapped using Tophat [32] to the complete Drosophila melanogaster 5.29 genome release available from Flybase. Given the size of these 36-base reads relative to the average exon length, a substantial fraction of reads will cover a splice junction. Hence, these reads will not align contiguously to the genome using standard read mapping methods. Tophat circumvents this problem by utilizing exon information to map reads across exon junctions. Tophat v1.0.14 was run with default parameters, in addition to allowing 3 segment mismatches and 1 splicing mismatch. When using Flybase annotations, a General Feature File (GFF file) was included with the "-G" and "--no-novel juncs" tags, ensuring that only known annotated exons were used. These mappings of full length reads and junction-reads were subsequently used by Cufflinks [42] to generate counts and coverages for annotated genes and their transcripts, all of whose annotations were retrieved from the 5.29 release of Flybase. Cufflinks v0.8.1 was run with default parameters with the following additional tags (-c 2 -F 0.05) and with the Flybase annotation included in a General Transcript File (GTF file).

To identify the set of genes expressed in Drosophila head tissues using *de novo *means with only the Drosophila genome sequence, all reads from every sample were pooled and mapped to the genome sequence. Tophat first mapped reads to the genome, identified potential exons, built a database of possible splice junctions, and then mapped the initially-unmapped reads against these junctions to confirm them. Cufflinks was used to assemble these annotations and their isoforms. Using the set of predicted junctions and annotations, sequenced reads from each sample were then analyzed independently, assigning reads to genes and isoforms that were identified *de novo*.

### TMM Normalization

Normalization among all samples were performed using the TMM protocol outlined in Robinson and Oshlack [55], which takes into account differences in overall RNA populations across biological samples and is one of several methods used to evaluate RNA sequencing data. The TMM normalization determined the ratio between all genes in one sample to another, removed the outliers beyond the ratio, and normalized using the modified ratio. Briefly, the TMM normalization calculates the log2 ratio of counts between two genotypes for all genes, and rescales the intensity of one genotype using the 30-70% quantile of these ratios. Normalization was implemented using the edgeR package in R [56]. Statistics and graphs evaluating consistency between replicates and genotypes were produced using the R statistical package.

Using these approaches, 8,561, 8,594, and 8,397 genes were expressed in female, male, and *tra*-mutant genotypes, respectively. Within a genotype, a gene was classified as expressed when all six replicates have FPKM values of at least 1. Data for expressed and non-expressed genes are provided in the Additional files.

### Statistical Identification of Differentially Expressed Genes and Transcripts

To identify annotations with differential expression between genotypes, the analysis procedure outlined in Marioni *et al*. [39] and modified in Robinson and Oshlack [55,57,58] were used. Briefly, this model assumed that the counts mapping to an annotation are Poisson-distributed and used an exact Poisson test for testing the differences between two genotypes. Similar to the Fisher's exact test, the probability of observing counts as or more extreme than observed was calculated and used to assess significance. The same likelihood ratio framework was then used to test for differences in expression between two genotypes. *q*-values were calculated by applying an FDR adjustment to account for multiple testing. The analysis was implemented using the edgeR package in R.

### Statistical Identification of Differential Isoform Usage

To identify genes under alternative splicing between genotypes, the number of sequence reads attributed to n isoforms for each gene was calculated. For replicates within genotypes, these counts were averaged to calculate:

where , , and  are the averages among six replicates for the read count of the nth isoform in the female, male and *tra*-mutant genotypes, respectively. A multinomial distribution was used to determine the likelihood of observing these counts given these fractions. A likelihood ratio test was used to test for differences in fractions between two genotypes (ie. H_0_: **F **= **M**). An FDR adjustment was made to account for multiple testing.

### DCC analysis

Genes associated with previously known DCC entry sites were identified, as follows. The DCC-bound regions include those found by ChIP-Chip and ChIP-seq studies [40,41]. From the ChIP-Chip study, 563 genes were reported, of which 514 genes were expressed in our study. From the ChIP-seq study, the physical chromosomal position of 805 peaks were reported, with 718 expressed in our study. Genes associated with these sites were identified by finding the nearest gene to the entry site, or the gene that the entry site resides within. This list of genes that reside next to a DCC-entry site was expanded by identifying additional genes within 10 kilobases of the DCC-entry site associated gene, or DCC-entry site physical map position, respectively.

### Gene Enrichment Analysis

All gene enrichment analyses for chromosome biases, and exon structural differences were performed using a Hypergeometric Test implemented in R, unless noted otherwise [R Development Core 59]. The Flymine web portal was used to assess GO functional enrichment. *P *values reported are based on the Benjamini and Hochberg test, implemented through Flymine [34].

## Authors' contributions

PLC took the lead on data analyses. JPD took the lead on generating the data. All authors conceived the experiments, contributed to the writing and all approve the submitted manuscript.

## Supplementary Material

Additional file 1 **Mapping statistics**. Mapping statistics for all technical replicates analyzed in the experiment, including number of reads, percent mapped, sequencing coverage. Click here for file

Additional file 2 **Genes covered in at least one genotype**. 9,473 genes covered in at least one genotype. Click here for file

Additional files 3 **Dot plots of FPKM between replicates for female, male and transformer RNA-seq data**. Dot plots of FPKM between replicates in log scale for female, male and transformer RNA-seq data. R^2 ^ranges between 0.93 and 0.96. Biological replicates are the following pairs 1 and 2, 3 and 4, and 5 and 6. Click here for file

Additional files 4 **Dot plots of FPKM between replicates for female, male and transformer RNA-seq data**. Dot plots of FPKM between replicates in log scale for female, male and transformer RNA-seq data. R^2 ^ranges between 0.93 and 0.96. Biological replicates are the following pairs 1 and 2, 3 and 4, and 5 and 6. Click here for file

Additional files 5 **Dot plots of FPKM between replicates for female, male and transformer RNA-seq data**. Dot plots of FPKM between replicates in log scale for female, male and transformer RNA-seq data. R^2 ^ranges between 0.93 and 0.96. Biological replicates are the following pairs 1 and 2, 3 and 4, and 5 and 6. Click here for file

Additional file 6 **Genes not covered any genotype**. 5,385 genes not covered in any genotype. Genes with no reads that map in any replicate are indicated in bold. Click here for file

Additional file 7 **Gene ontology functional analysis of genes not covered in any genotype**. Gene ontology functional analysis of 5,385 that are not covered in any genotype. Click here for file

Additional file 8 **Chromosome distribution bias for genes**. Chromosome distribution bias for genes expressed, genes not covered, genes differentially expressed, and genes alternatively spliced. Boldface indicate significantly over- (blue) or underrepresented (red) based on the total number of genes in the available pool, based on a one-sided Hypergeometric Test. Click here for file

Additional file 9 **Average coverage distribution**. Average coverage distribution along annotation unit for all gene transcripts covered, shown from 5' (left) to 3' (right) for female (red), male (blue), and tra pseudomale (black) genotypes. Introns were removed. Transcripts were broken into 50 equal-length regions and each region was normalized based on the coverage of the entire transcript. Click here for file

Additional file 10 **Genes expressed in at least one genotype**. 8,896 genes expressed in at least one genotype. Red indicate genes with female-biased expression. Blue indicate genes with male-biased expression. Click here for file

Additional file 11 **Genes expressing more than 1 isoform**. 1,722 genes expressing more than 1 isoform. Bold indicate genes downstream of *tra*. Click here for file

Additional file 12 **Comparison of junction coverage with transcript isoform FPKM**. Comparison of junction coverage with transcript isoform FPKM, for female (R^2 ^= 0.70), male (R^2 ^= 0.73), and *tra *pseudomale (R^2 ^= 0.74) genotypes. Click here for file

Additional file 13 **Isoforms expressed among 1,722 genes**. 4,368 isoforms expressed among 1,722 genes. Red indicate genes with female-biased expression. Blue indicate genes with male-biased expression. Click here for file

Additional file 14 **Average coverage distribution of constitutive introns**. Average coverage distribution of constitutive introns along full annotation unit for 1,722 genes expressing more than one isoform transcript, shown from 5' (left) to 3' (right) for female (red), male (blue), and *tra *pseudomale (black) genotypes. Each gene was broken into 50 equal-length regions and each region was normalized based on the coverage of the entire gene. Click here for file

Additional file 15 **Average coverage distribution of constitutive exons**. Average coverage distribution of constitutive exons along full annotation unit for 1,722 genes expressing more than one isoform transcript, shown from 5' (left) to 3' (right) for female (red), male (blue), and *tra *pseudomale (black) genotypes. Each gene was broken into 50 equal-length regions and each region was normalized based on the coverage of the entire gene. Click here for file

Additional file 16 **Average coverage distribution of cassette exons**. Average coverage distribution of cassette exons along full annotation unit for 1,722 genes expressing more than one isoform transcript, shown from 5' (left) to 3' (right) for female (red), male (blue), and *tra *pseudomale (black) genotypes. Each gene was broken into 50 equal-length regions and each region was normalized based on the coverage of the entire gene. Click here for file

Additional file 17 **Average fold-change between female and male for constitutive exons, cassette exons, and constitutive introns**. Average fold-change between female and male for constitutive exons, cassette exons, and constitutive introns along full annotation unit for 1,722 genes expressing more than one isoform transcript, shown from 5' (left) to 3' (right). Each gene was broken into 50 equal-length regions and each region was normalized based on the coverage of the entire gene. Click here for file

Additional file 18 **Coverage distribution of constitutive exons, cassette exons, and constitutive introns of varying lengths for genes expressing more than one isoform transcript**. Coverage distribution of constitutive exons, cassette exons, and constitutive introns of varying lengths for 1,722 genes expressing more than one isoform transcript for female (red) and male (blue). Click here for file

Additional file 19 **Average fold-change between female and male for constitutive exons, cassette exons, and constitutive introns of varying lengths for genes expressing more than one isoform transcript**. Average fold-change between female and male for constitutive exons, cassette exons, and constitutive introns of varying lengths for 1,722 genes expressing more than one isoform transcript. Click here for file

Additional file 20 **Genes with Tra binding sites**. 10 genes with Tra binding sites. Click here for file

Additional file 21 **Isoforms for genes that have the Tra binding site**. Isoforms for the 10 genes that have the Tra binding site. It should be noted that the location of the Tra binding site relative to each isoform is not shown. Click here for file

Additional file 22 **Genes identified *de novo***. 169 genes found between known annotated genes (NCUFF genes). 651 genes found overlapping previously annotated genes are also included (CUFF genes). Click here for file
