# Supplementary figures and images for: Somatic sex-specific transcriptome differences in Drosophila revealed by whole transcriptome sequencing
Source: BMC Genomics. 2011 Jul 14;12:364. doi: 10.1186/1471-2164-12-364 (PMC3152543; doi:10.1186/1471-2164-12-364)

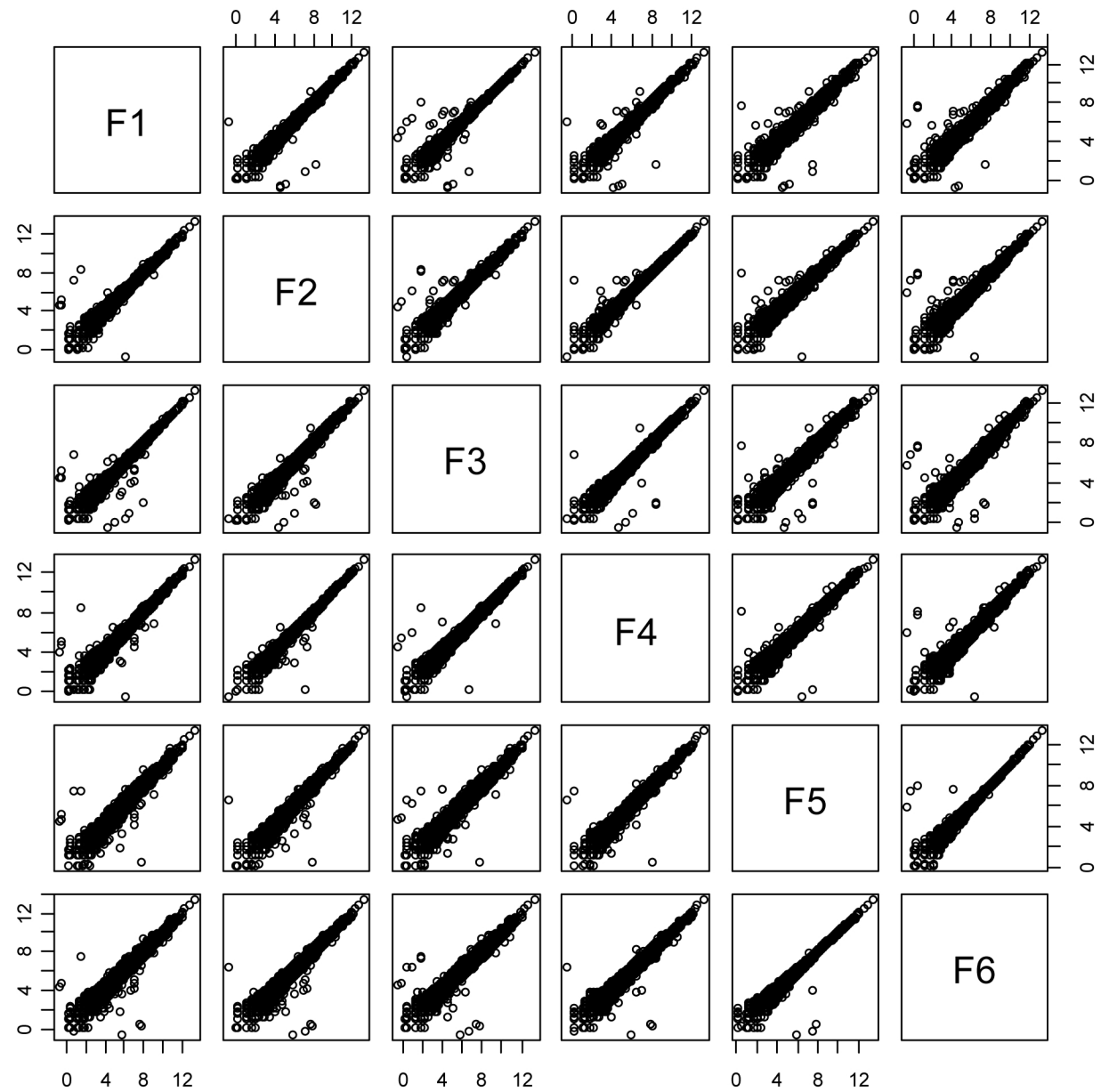

Supplement: Additional files 3 — Dot plots of FPKM between replicates for female, male and transformer RNA-seq data. Dot plots of FPKM between replicates in log scale for female, male and transformer RNA-seq data. R2 ranges between 0.93 and 0.96. Biological replicates are the following pairs 1 and 2, 3 and 4, and 5 and 6. [file 1471-2164-12-364-S3.PDF]

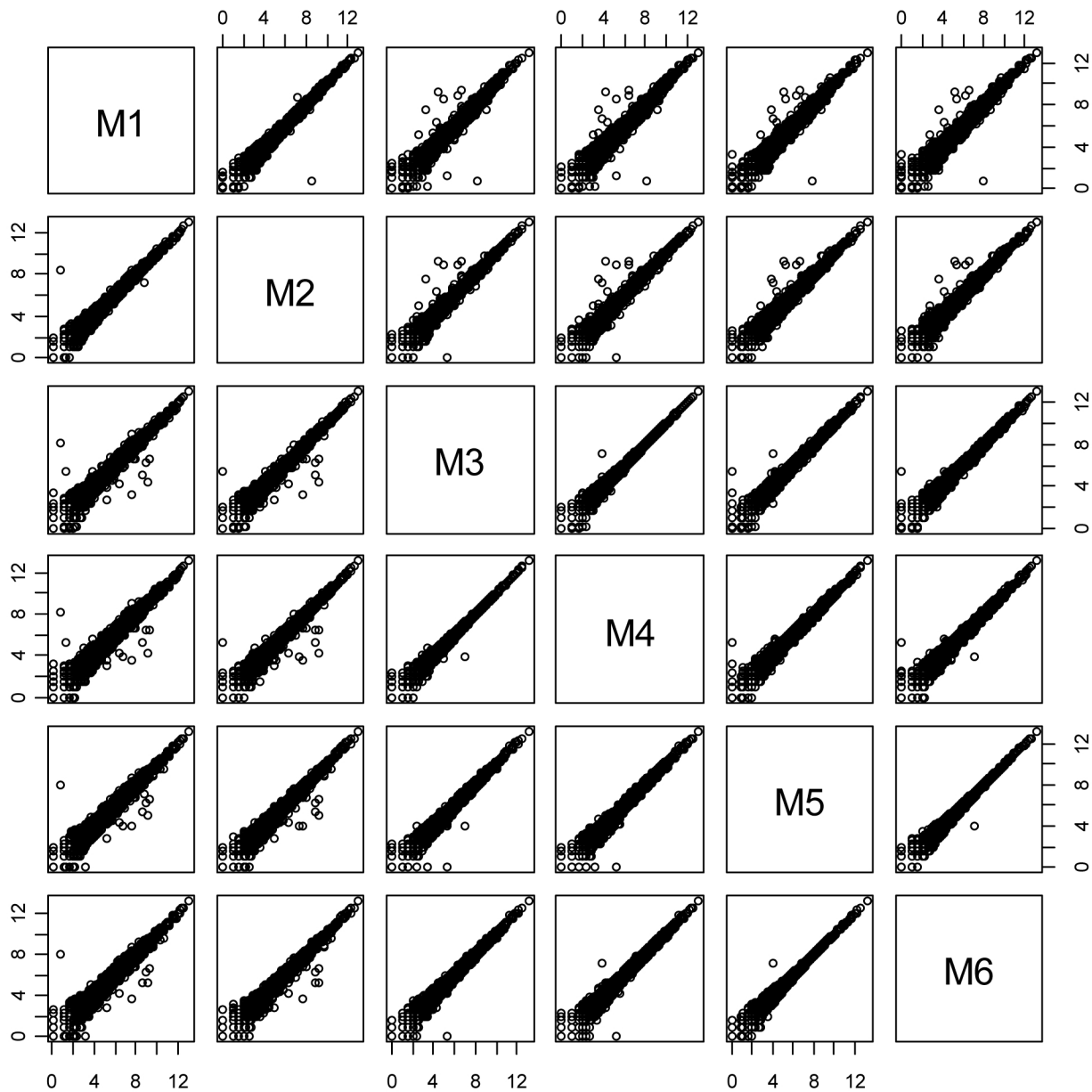

Supplement: Additional files 4 — Dot plots of FPKM between replicates for female, male and transformer RNA-seq data. Dot plots of FPKM between replicates in log scale for female, male and transformer RNA-seq data. R2 ranges between 0.93 and 0.96. Biological replicates are the following pairs 1 and 2, 3 and 4, and 5 and 6. [file 1471-2164-12-364-S4.PDF]

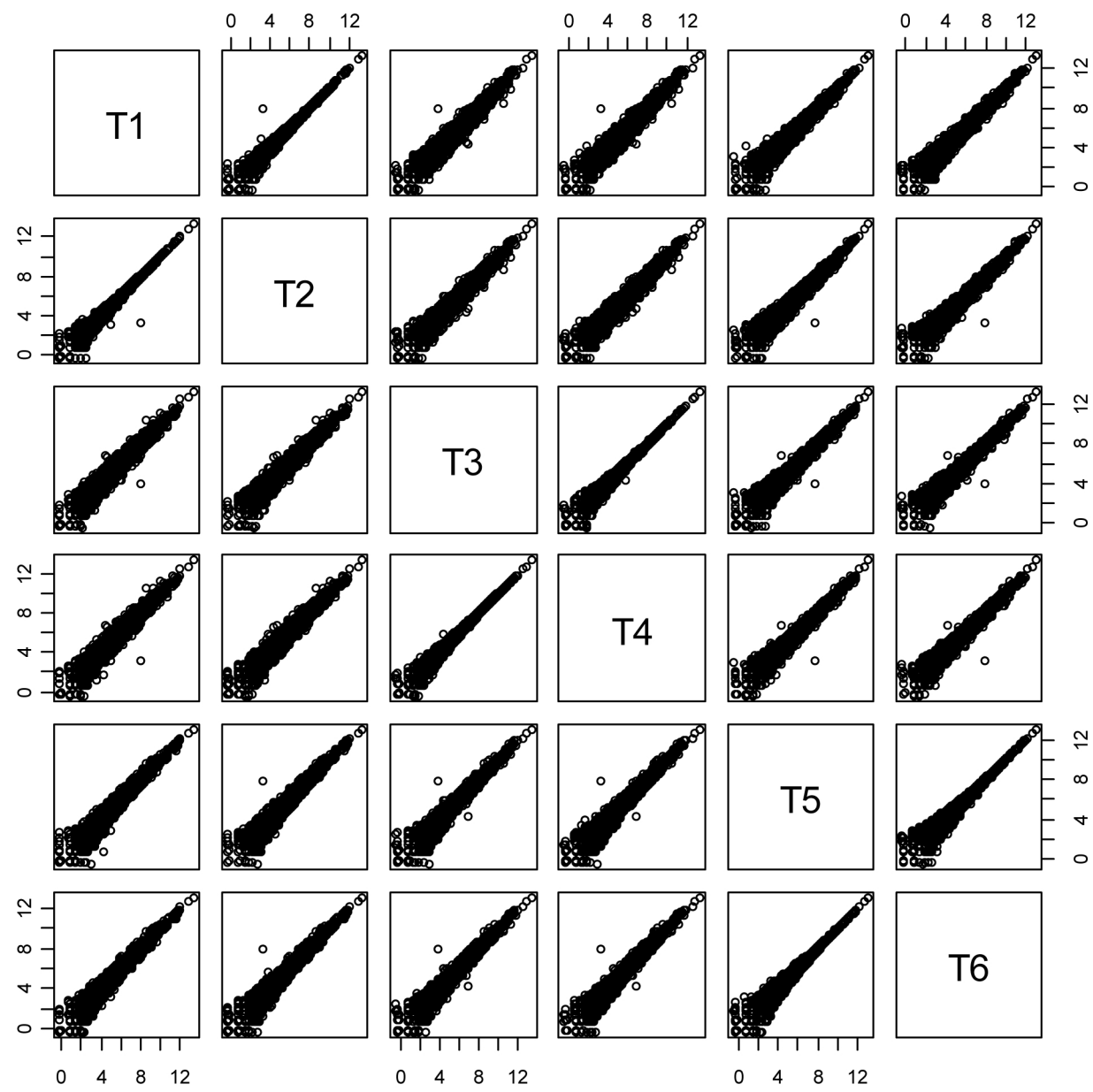

Supplement: Additional files 5 — Dot plots of FPKM between replicates for female, male and transformer RNA-seq data. Dot plots of FPKM between replicates in log scale for female, male and transformer RNA-seq data. R2 ranges between 0.93 and 0.96. Biological replicates are the following pairs 1 and 2, 3 and 4, and 5 and 6. [file 1471-2164-12-364-S5.PDF]

Additional File 9

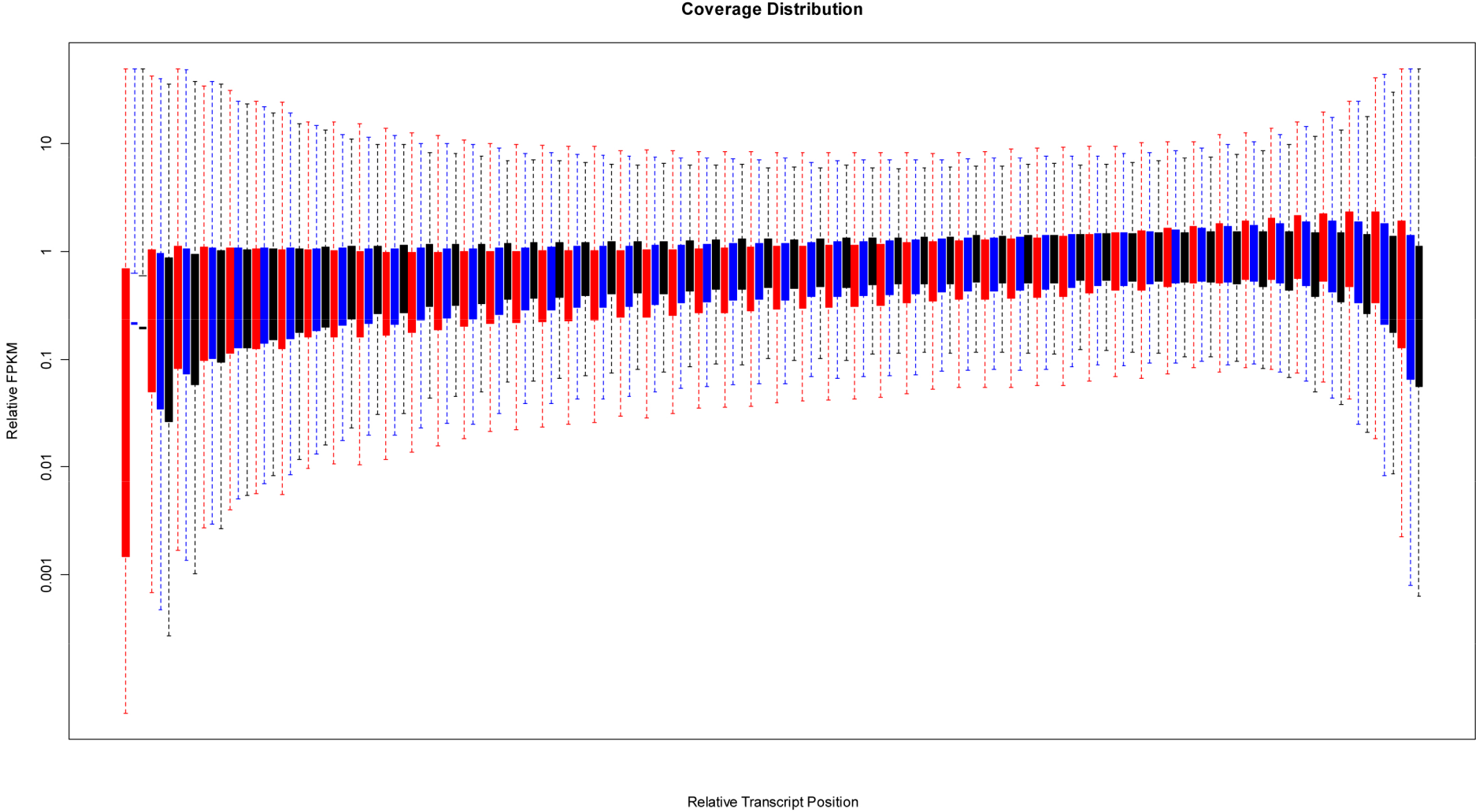

Supplement: Additional file 9 — Average coverage distribution. Average coverage distribution along annotation unit for all gene transcripts covered, shown from 5' (left) to 3' (right) for female (red), male (blue), and tra pseudomale (black) genotypes. Introns were removed. Transcripts were broken into 50 equal-length regions and each region was normalized based on the coverage of the entire transcript. [file 1471-2164-12-364-S9.PDF]

**Female Junctions**

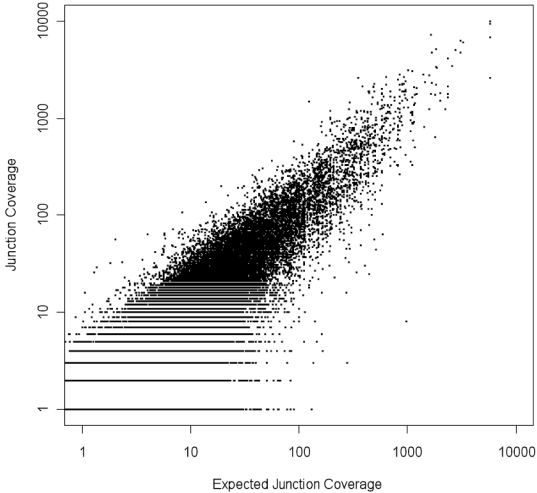

**Male Junctions**

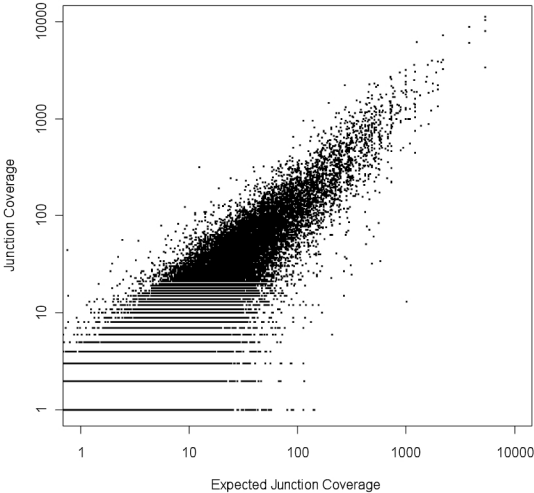

**Pseudomale Junctions**

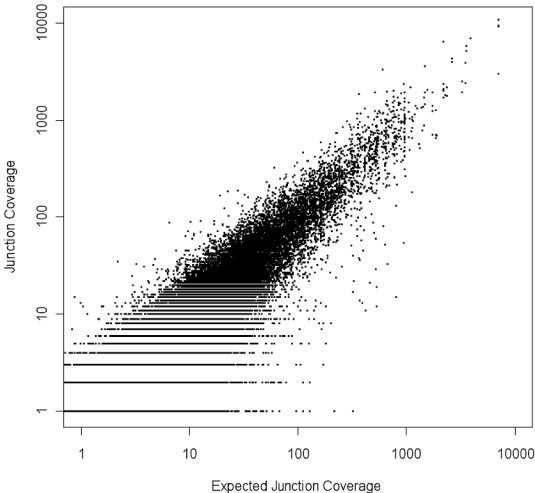

Supplement: Additional file 12 — Comparison of junction coverage with transcript isoform FPKM. Comparison of junction coverage with transcript isoform FPKM, for female (R2 = 0.70), male (R2 = 0.73), and tra pseudomale (R2 = 0.74) genotypes. [file 1471-2164-12-364-S12.PDF]

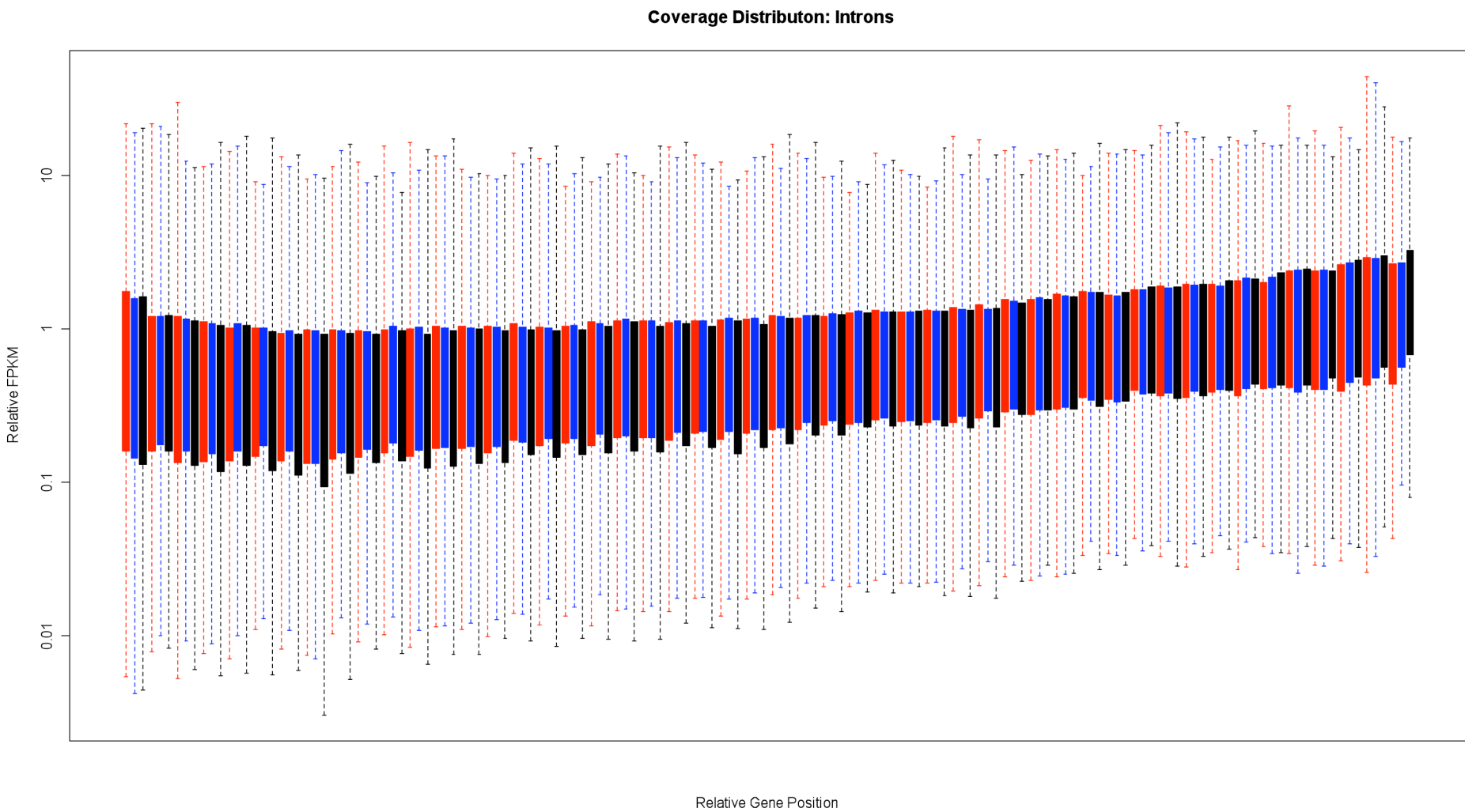

Supplement: Additional file 14 — Average coverage distribution of constitutive introns. Average coverage distribution of constitutive introns along full annotation unit for 1,722 genes expressing more than one isoform transcript, shown from 5' (left) to 3' (right) for female (red), male (blue), and tra pseudomale (black) genotypes. Each gene was broken into 50 equal-length regions and each region was normalized based on the coverage of the entire gene. [file 1471-2164-12-364-S14.PDF]

Coverage Distributon: Constitutive Exons

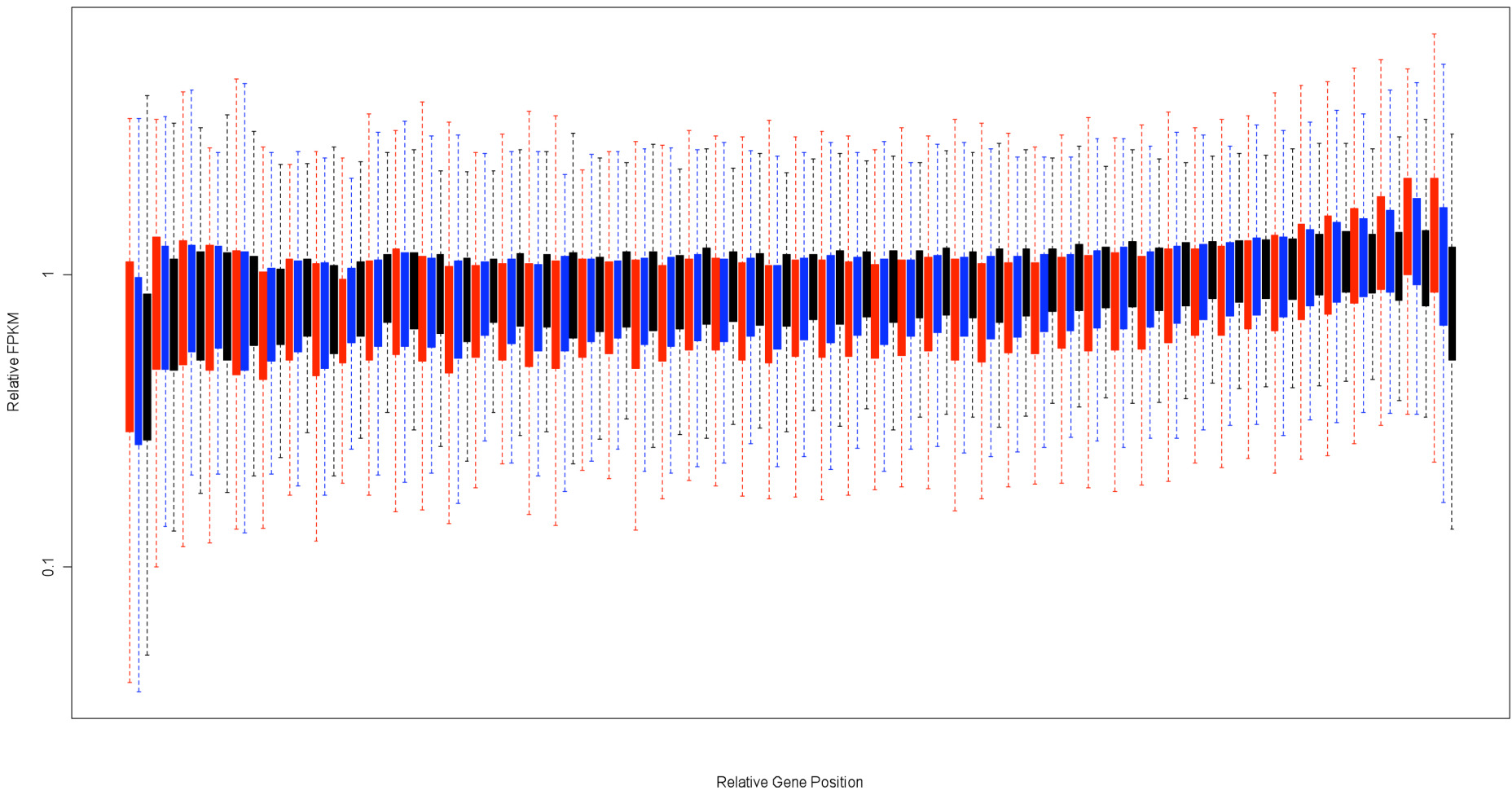

Supplement: Additional file 15 — Average coverage distribution of constitutive exons. Average coverage distribution of constitutive exons along full annotation unit for 1,722 genes expressing more than one isoform transcript, shown from 5' (left) to 3' (right) for female (red), male (blue), and tra pseudomale (black) genotypes. Each gene was broken into 50 equal-length regions and each region was normalized based on the coverage of the entire gene. [file 1471-2164-12-364-S15.PDF]

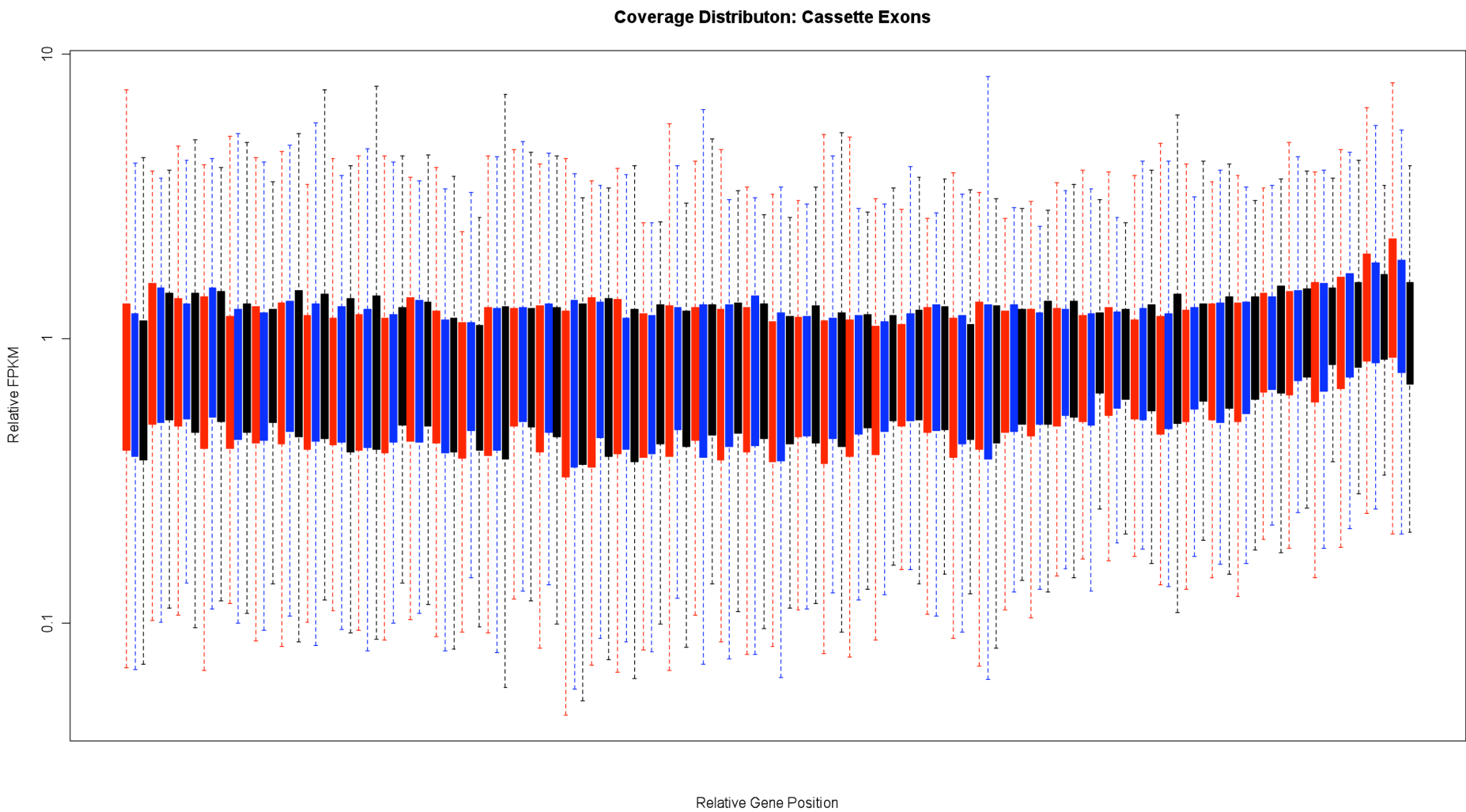

Supplement: Additional file 16 — Average coverage distribution of cassette exons. Average coverage distribution of cassette exons along full annotation unit for 1,722 genes expressing more than one isoform transcript, shown from 5' (left) to 3' (right) for female (red), male (blue), and tra pseudomale (black) genotypes. Each gene was broken into 50 equal-length regions and each region was normalized based on the coverage of the entire gene. [file 1471-2164-12-364-S16.PDF]

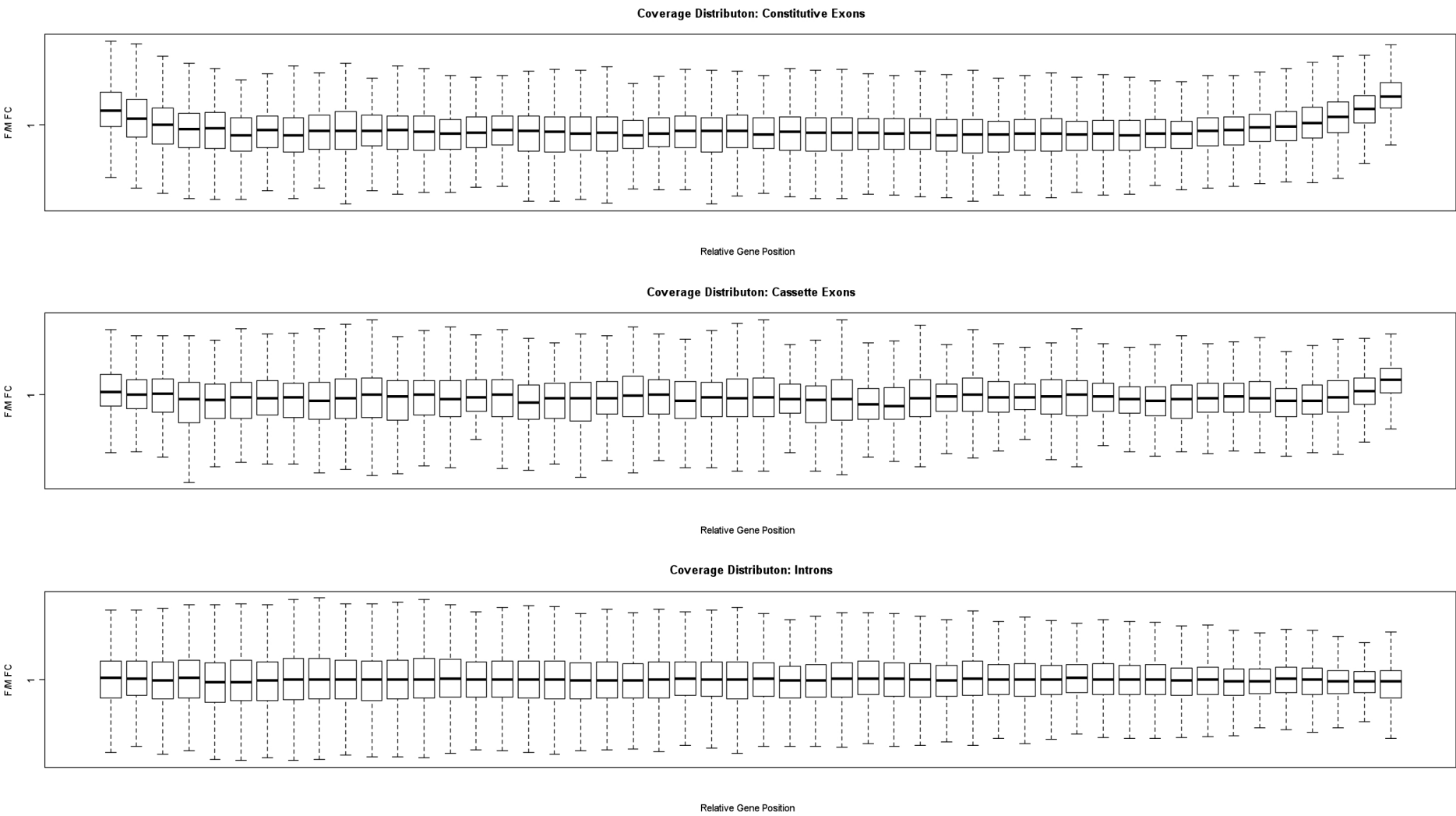

Supplement: Additional file 17 — Average fold-change between female and male for constitutive exons, cassette exons, and constitutive introns. Average fold-change between female and male for constitutive exons, cassette exons, and constitutive introns along full annotation unit for 1,722 genes expressing more than one isoform transcript, shown from 5' (left) to 3' (right). Each gene was broken into 50 equal-length regions and each region was normalized based on the coverage of the entire gene. [file 1471-2164-12-364-S17.PDF]

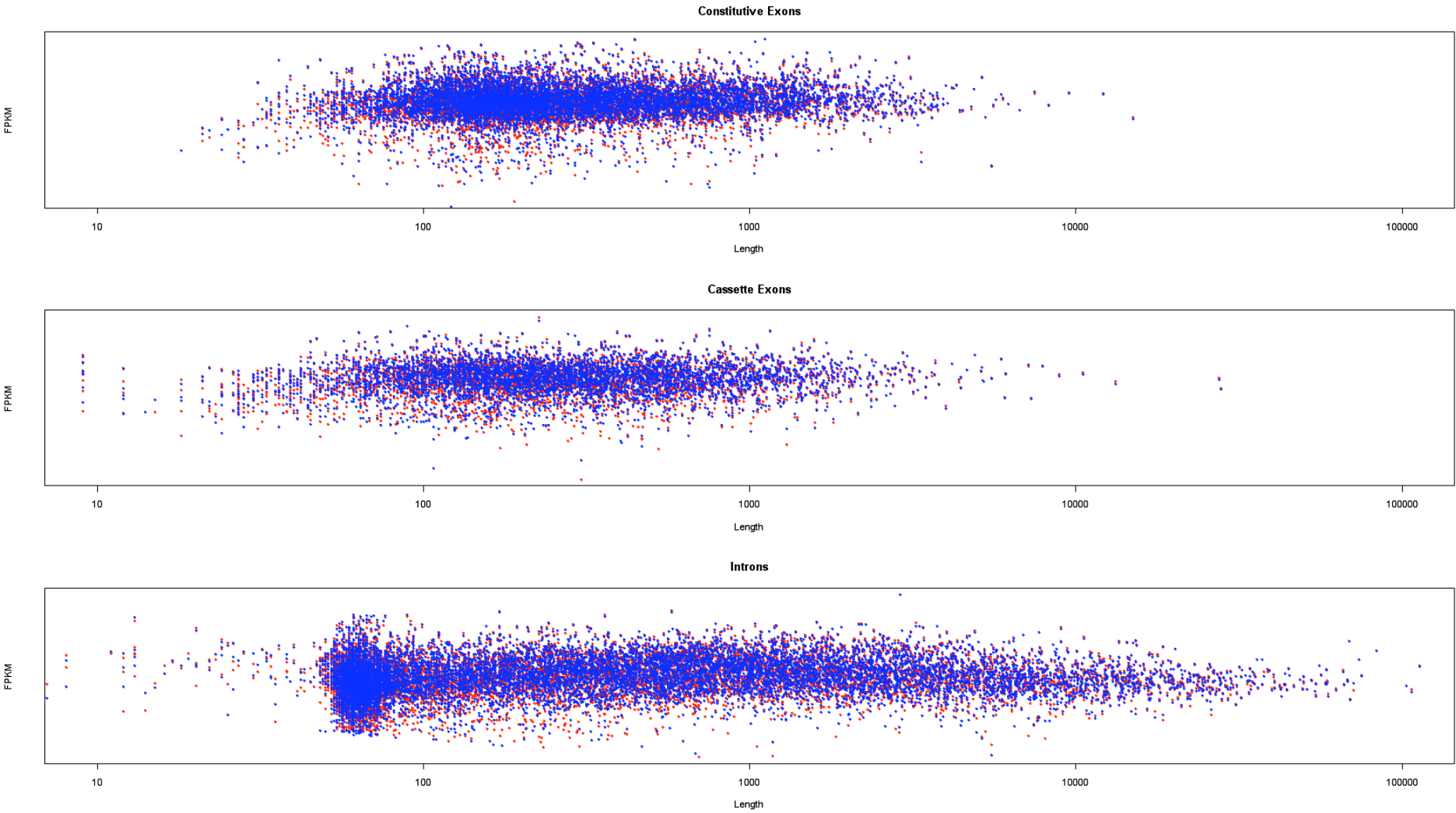

Supplement: Additional file 18 — Coverage distribution of constitutive exons, cassette exons, and constitutive introns of varying lengths for genes expressing more than one isoform transcript. Coverage distribution of constitutive exons, cassette exons, and constitutive introns of varying lengths for 1,722 genes expressing more than one isoform transcript for female (red) and male (blue). [file 1471-2164-12-364-S18.PDF]

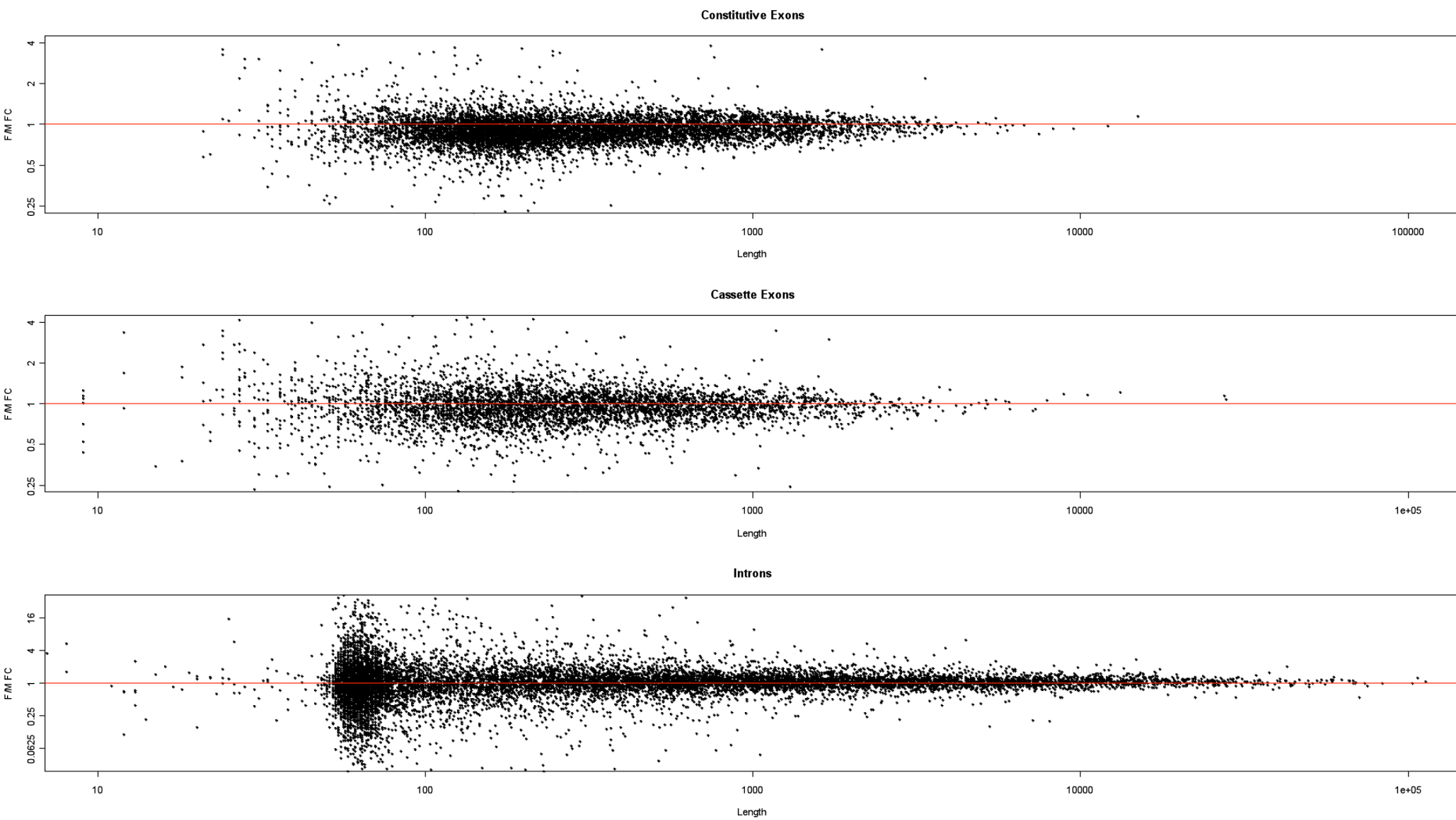

Supplement: Additional file 19 — Average fold-change between female and male for constitutive exons, cassette exons, and constitutive introns of varying lengths for genes expressing more than one isoform transcript. Average fold-change between female and male for constitutive exons, cassette exons, and constitutive introns of varying lengths for 1,722 genes expressing more than one isoform transcript. [file 1471-2164-12-364-S19.PDF]
